# Canine Olfactory Detection of a Non-Systemic Phytobacterial Citrus Pathogen of International Quarantine Significance

**DOI:** 10.3390/e22111269

**Published:** 2020-11-09

**Authors:** Timothy Gottwald, Gavin Poole, Earl Taylor, Weiqi Luo, Drew Posny, Scott Adkins, William Schneider, Neil McRoberts

**Affiliations:** 1 U.S. Department of Agriculture, Agricultural Research Service, Fort Pierce, FL 34945, USA; gavin.poole@usda.gov (G.P.); earl.taylor@usda.gov (E.T.); weiqi.luo@usda.gov (W.L.); Drew.posny@usda.gov (D.P.); scott.adkins@usda.gov (S.A.); 2 Center for Integrated Pest Management, North Carolina State University, Raleigh, NC 27695, USA; 3 F1K9 LLC, Palm Coast, FL 34797, USA; wschneider@f1-k9.com; 4 Plant Pathology Department, University of California Davis, Davis, CA 95616, USA; nmcroberts@ucdavis.edu

**Keywords:** early detection, Asiatic citrus canker, latent class, information theory, field diagnostic, scent signature, direct assay, deployment

## Abstract

For millennia humans have benefitted from application of the acute canine sense of smell to hunt, track and find targets of importance. In this report, canines were evaluated for their ability to detect the severe exotic phytobacterial arboreal pathogen *Xanthomonas citri* pv. *citri* (Xcc), which is the causal agent of Asiatic citrus canker (Acc). Since Xcc causes only local lesions, infections are non-systemic, limiting the use of serological and molecular diagnostic tools for field-level detection. This necessitates reliance on human visual surveys for Acc symptoms, which is highly inefficient at low disease incidence, and thus for early detection. In simulated orchards the overall combined performance metrics for a pair of canines were 0.9856, 0.9974, 0.9257 and 0.9970, for sensitivity, specificity, precision, and accuracy, respectively, with 1–2 s/tree detection time. Detection of trace Xcc infections on commercial packinghouse fruit resulted in 0.7313, 0.9947, 0.8750, and 0.9821 for the same performance metrics across a range of cartons with 0–10% Xcc-infected fruit despite the noisy, hot and potentially distracting environment. In orchards, the sensitivity of canines increased with lesion incidence, whereas the specificity and overall accuracy was >0.99 across all incidence levels; i.e., false positive rates were uniformly low. Canines also alerted to a range of 1–12-week-old infections with equal accuracy. When trained to either Xcc-infected trees or Xcc axenic cultures, canines inherently detected the homologous and heterologous targets, suggesting they can detect Xcc directly rather than only volatiles produced by the host following infection. Canines were able to detect the Xcc scent signature at very low concentrations (10,000× less than 1 bacterial cell per sample), which implies that the scent signature is composed of bacterial cell volatile organic compound constituents or exudates that occur at concentrations many fold that of the bacterial cells. The results imply that canines can be trained as viable early detectors of Xcc and deployed across citrus orchards, packinghouses, and nurseries.

## 1. Introduction

Asiatic citrus canker (ACC) is a fruit, foliar, and twig lesion disease that has significant international, national, and local quarantine implications and has been the focus of multiple extensive eradication programs. The disease is caused by the bacterium *Xanthomonas citri* pv. *citri* (Xcc). When pathogen infection is severe, leaf drop and up to 69% crop loss can occur due to fruit drop [1]. The bacteria disperse via meteorological events ranging from gentle rain to tropical storms and hurricanes. The more severe the meteorological event, the more effective the inoculum dispersal [2,3]. Hurricanes and tropical storms have been associated with long-range dissemination as well as local increase of the bacterium [2,4,5,6]. Xcc inoculum can also be transmitted mechanically by machinery and humans if the foliage is wet. Inoculum begins to exude from Xcc lesions within 1–5 min of becoming wet [4,6,7,8]. The maximum concentration of bacteria is exuded within the first 1–2 h period following the beginning of the rainfall event or wetting of the lesion, although inoculum is produced continuously at a lower concentration for the duration of the wetting period or storm [2,3,4]. Infection takes place when inoculum-laden water passes through stomata of foliage, fruit, or green twigs. Infection can occur through wounds as well, and it is highly exacerbated by the Asian citrus leafminer (*Phyllocnistis citrella* Stainton), whose feeding galleries create a labyrinth of wounds that expose susceptible leaf mesophyll tissues to splashed inoculum, greatly increasing the probability of infection by Xcc [9,10].

After infection occurs, bacteria propagate within the plant tissues, eventually forming small blister-like protrusions that become visible 5–7 days post-infection with close examination augmented by 10× magnification. Within 12–14 days, these protrusions erupt through the epidermis, forming 1–2 mm light brown erumpent lesions. As the lesions age, they darken to brown and develop water-soaked margins with a surrounding chlorotic halo [7]. The lesion center develops a raised spongy and corky appearance on adaxial and abaxial foliar surfaces. Older lesions can reach 1 cm in diameter and can coalesce to form mass infections.

Pathogen detection in the field is almost exclusively by human visual inspection. Confirmation of Xcc infection can be accomplished by serological or Polymerase Chain Reaction (PCR) assays, both of which require infected tissue. Therefore, such assays are of minimal use for a field survey of a pathogen that causes only local lesions, i.e., non-systemic infections. Visual inspection is tedious, labor intensive, and highly variable due to the elusiveness of Xcc lesions, especially when high in the canopy. Since mature citrus trees can have in excess of 100,000 leaves, finding initial infections visually in such a large canopy is challenging and uncertain. As a consequence, multiple infection cycles must occur before symptoms are of sufficient prevalence to permit visual detection. During a major epidemic in Florida (1996–2006), the detection time for Xcc by trained regulatory inspectors averaged 106 days post-infection [11]. In one regulatory exercise, 14 teams of two inspectors per team examined the same infected orchard in succession. No two teams found the same infected trees, each successive team found new infected trees previously undetected by prior teams, and no team found all known infections. [Riley, unpublished results]. Since it is highly improbable that all infected trees in an orchard will be found by visual inspection, the true incidence of infection cannot be determined, and thus, calculating the detection accuracy of visual inspection is not possible.

The unpromising results for human visual detection suggest the need for the deployment of a detection technology with much better performance. Genetic analyses indicate that modern-day canines (*Canis lupus familiaris*) were the first domesticated animal, arising from two separate wolf (*Canis lupus*) populations ≈ 15,000 years ago [12,13,14,15,16,17]. The mammalian (including canids) olfactory system is antediluvian, having evolved from early chemotactic receptors during the Precambrian over 600 million years ago. Acute olfaction evolved to enhance finding food, mates, detecting danger, avoiding predators, etc. [18]. An overarching advantage to the human use of canine olfaction is that all canids non-destructively interrogate their environment for a scent signature of interest. This scent signature is composed of a specific volatile organic compound (VOC) or complex of VOCs. In contrast, commonly used molecular or biochemical assays often require destructively sampling a small proportion of the host or environment and are specific and do not detect complex VOC composites.

Wolves employ acute vision and hearing when prey is in close proximity. However, when tracking prey, often over great distances, wolves resort to olfactory cues to locate widely dispersed and often low-density prey [19], as is true for domestic canines [20]. To find rare targets of human interest, we exploit the tracking expertise and acute sense of smell handed down to modern canines from wolves and their evolutionary predecessors. Nonetheless, scent detection is pervasive through the animal kingdom. Other vertebrates and invertebrate animals have been explored recently in the burgeoning science of scent detection research with diverse practical applications [21]. However, canines dominate as olfactory detector tools because of their unsurpassed domestication and easily deployed tracking skills.

Canines are used broadly to detect and locate a wide array of organic and inorganic odors, (e.g., explosives, drugs, accelerants, pollutants, toxins, pesticides), tracking humans and game animals, finding cadavers, and matching the scent of criminal perpetrators to crime scenes [22]. Canines are also proficient at medical human disease detection, especially cancer (malignant melanoma, non small-cell, small-cell lung, breast, prostrate, bladder, ovarian, and colorectal cancers) often with equivalent or superior sensitivity to current medical assays and can detect epileptic seizures prior to onset [22,23,24,25].

Relative to agricultural diseases, canines were recently trained extensively to detect *Candidatus* Liberibacter asiaticus (CLas), which is the causal pathogen of citrus Huanglongbing with greater than 99%, 96%, and 92% accuracy in field trials, commercial citrus orchards and citrus in residential properties, respectively [26]. In the same study, canines detected CLas in the Asian citrus psyllid, *Diaphorina citri,* that vectors the bacteria and in bacterial co-cultures devoid of plant or animal host cells. Additionally, canines have been trained to detect plum pox virus in commercial peach (*Prunus persica*) orchards [Gottwald unpublished] and the fungal pathogen *Raffaelea lauricola*, which is the cause of Laurel wilt disease of avocado (*Persea americana*) [27]. Thus, canines have been demonstrated to detect bacterial, viral, and fungal pathogens in plant hosts.

Canine detection of the plant pathogens as indicated above represents a novel extension of previous detection targets. For drugs, explosives, pollutants, etc., canines are trained to detect specific VOC scent signatures of the target compounds. For biologicals such as plants, plant parts, humans, and other animals, the canines are trained to recognize the volatilome, e.g., unique VOC complex, emitted by the target. Conversely, when trained to detect pathogenic organisms, the canines must detect one organism (bacterium, virus, or fungus) within another organism, i.e., the host plant or animal. The three plant pathogens discussed above, CLas, plum pox virus and *R. lauricola,* are all systemic within the vascular system of the host, and therefore, they can be completely or incompletely distributed within the plant as the infection progresses. In contrast, Xcc is non-systemic and causes only local lesions, which can range in incidence from a single lesion to thousands per tree and can be rare, highly aggregated, or diffused within the host.

In this study, we demonstrate proof of concept that canines can discriminate and detect Xcc and can be trained as viable detectors of the pathogen in agricultural environments. Here we document the detection of Xcc, an exotic bacterial pathogen by use of canine olfactory surveillance in orchards and packinghouses. Finally, we determine that canines can directly detect the target bacterium *in planta* and *in vitro*.

## 2. Materials and Methods

### 2.1. Initial Sensitization Training of Canines for Xcc Scent Signature Recognition

Eleven canines of various breeds were utilized for the various trials throughout the course of the study (Table 1).

**Figure 1:**
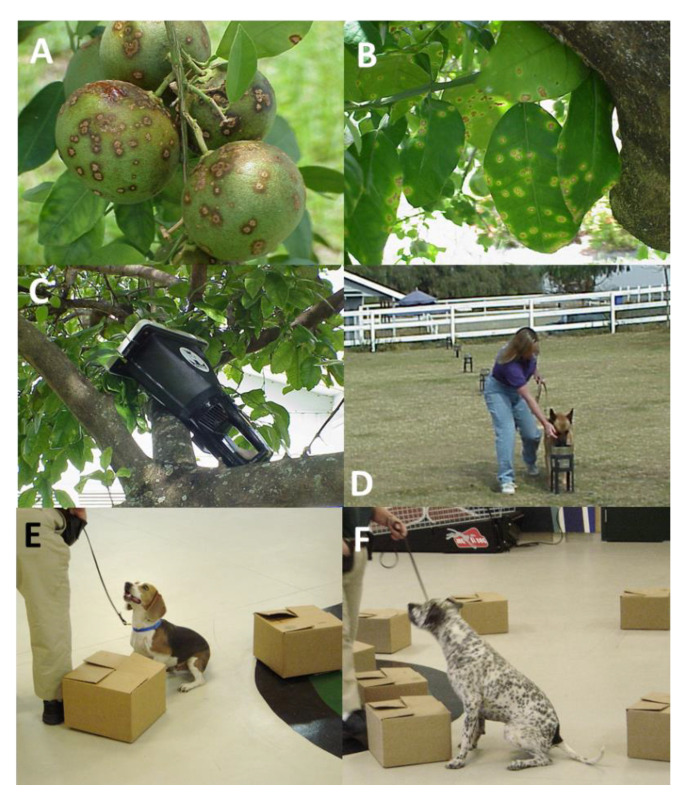
Initial training of canines for detection of the phytobacterial arboreal pathogen *Xanthomonas citri* pv. *citri* (Xcc), the causal agent of Asiatic citrus canker (Acc). (**A**) Xcc-infected red grapefruit fruit and (**B**) foliage. (**C**) Scent Transfer Unit (STU) used to draw in Xcc volatiles and deposit on cotton scent collection pad. (**D**) Canine “Kimba” training by interrogating a row of metal cans containing Xcc-positive and negative scent pads. (**E**,**F**) Detector canine NDD-1 and NDD-3 alerting on boxes containing Xcc-infected foliage at the USDA, APHIS, National Detector Dog Training Center.

At the onset of this study, Xcc was considered a quarantine pathogen in Florida [3,6,7,11], and neither the bacteria nor infected plant material could be transported to new locations for experimental purposes. Therefore, we used a Scent Transfer Unit (STU) (Model STU-100, Tolhurst Big “T” Enterprises, Lockport, New York, NY, USA, 14094) vacuum device commonly used in canine detection work to draw air at a constant rate (≈300 L/min) to collect volatile samples containing the “scent signature” of the target, in this case Xcc-infected plants, and deposit the scent onto a cotton “scent pad” [28]. Since the scent pad does not contain the pathogen (Xcc is only splash dispersed, generally by rain and not dispersed in dry air) nor any potentially infected plant material, it can be safely transported to non-endemic areas for testing. Scent pad samples were collected from non-infected and Xcc-infected Ruby Red grapefruit (*Citrus paradisii*) trees using the STU. A sterile 12.7 × 22.9 cm (5 × 9 inch) cotton pad was placed into the STU for 10 min by setting the STU within the canopy of a non-infected or Xcc-infected tree 5–10 cm from leaves, fruit and branches while air was pulled through the pad. Following each 10 min sampling period, scent pads were aseptically removed, placed in a volatile-proof plastic bag (K-pak), and heat sealed. The STU was disinfected with ethyl alcohol between each use. Multiple heat-sealed samples from infected and non-infected trees were segregated, placed in zip lock plastic bags, and stored at −20 °C until transported (Figure 1A–C).

Canine detection companies are for-profit ventures, and therefore, they are often reticent to undertake non-profitable and time-consuming basic research. Fortuitously, a canine detection company in California was willing to dedicate two canines to do an initial testing of canine detection of Xcc. In preliminary studies, it was not feasible to bring canines to the Xcc-infected sites due to quarantine constraints. Therefore, scent pad samples were collected in South Florida, sealed in a styrofoam shipping box with ice packs, and transported overnight to the California training facility.

One Belgian Malinois (Kimba) and one German Shepard (Tank), previously trained for criminology scent detection, were trained to the Xcc scent signature by imprinting, during which the canine was introduced to the target and other neutral scents. Relative to scent signature training, when the canine becomes interested in/reacts to the correct target, the experience is immediately encouraged with verbal and play rewards [18]. In our case, canines were imprinted to the scent signature presented on Xcc-infected scent pads to discriminate the scent from scent pads without the scent signature. To take advantage of the candid passion to search and track, a mixed population of mostly non-infected with a few Xcc-infected scent pad samples were arrayed in a series of rows of metal cans placed outdoors on metal stands (Figure 1D). Correctly alerting on the Xcc-infected pad placed in the bottom of a can resulted in the canine receiving verbal praise and a few moments of play with the handler and a ball or “kong” (hard rubber ball on a short rope). Initial training used non-frozen scent pads. Canines subsequently detected both non-frozen and frozen scent pads from Xcc-infected trees. Thereafter, scent pads were stored frozen until use in training and assessment.

Following initial scent training, a series of preliminary studies were conducted in which canines interrogated scent pads exposed to Xcc-infected red grapefruit for periods of 1, 5, 10, or 30 min versus pads exposed to non-infected trees for similar times, to determine if there was a lower threshold of exposure necessary to create scent pad training materials. Additionally, once the scent pads were removed from the heat-sealed plastic bags, the canines interrogated them every few days to determine the temporal viability of the scent pads as a training tool.

### 2.2. Training Canines for Detection of Xcc-Infected Plant Material

During 2004–5, in collaboration with the U.S. Department of Agriculture, Animal and Plant Health Inspection Service (USDA, APHIS), National Detector Dog Training Center (NDDTC) in Orlando, Florida, we trained canines to detect Xcc-infected Duncan grapefruit seedlings. At the USDA, ARS laboratory in Fort Pierce, Florida, quarantine greenhouse, seedlings were infected by placing drops of Xcc inoculum, ≈10^6^ to 10^8^ cfu/mL prepared from pure cultures on young leaves and using a sterile needle to wound the leaf lamina, causing 5–10 wounds/leaf of five leaves/seedling. Seedlings were incubated in the greenhouse for ≈2–3 weeks until symptomatic and then transported under regulatory permit in sealed containers to the NDDTC in Orlando along with non-infected seedlings as training materials where they were maintained until use. At the NDDTC, two beagles (NDD-1 and NDD-2), one German pointer (NDD-3), and one Labrador retriever (NDD-4) were trained to recognize the Xcc scent signature by a similar imprinting/reward method as described above. Scent detection proficiency was tested by placing excised branches or whole potted Xcc-infected or non-infected trees in closed cardboard boxes and arraying the boxes on the floor of the training facility (Figure 1E,F). In a second NDDTC study, the trained Labrador was transported to an Xcc-infected orchard in Indian River County, Florida. To avoid disease quarantine issues, leaves were collected from Xcc-infected and non-infected trees and placed into separate sealed plastic pipe containers with holes drilled in the sides to allow the volatiles but not infected plant material to escape for canine interrogation. The canine interrogated the containers, which were placed on the ground at the edge of the orchard. When the trial ended, the infected and non-infected plant materials were removed from the containers, discarded in the infected field, and the containers were disinfected. Due to quarantine issues, intermittent and uncoordinated testing by NDDTC personnel in absence of the authors, and uncoordinated replacement of canines assigned to the study, data were not consistently taken with the exception of an overall assessment of performance by NDDTC personnel.

In all subsequent studies, we collaborated with three commercially certified canine detector companies who specialize in trained detector canines for military, police and domestic clientele, for the detection of an array of targets including explosives, drugs, bed bugs, etc. This was important because professional canine handlers are themselves trained not to give any voluntary or involuntary cues to the canines while testing their performance. A single handler was used for all replications of most studies to avoid bias or variation due to handler. These companies trained nine canines (7 were used in this study) for the detection of Xcc via imprinting on scent pads from Xcc-infected trees or Xcc-infected grapefruit seedlings until canines gained proficiency at differentiating Xcc-infected from non-infected samples (Table 1).

### 2.3. Assessment of Canine Performance

To measure the performance of canines individually and as a group, a binary classification test was performed on the data from each of the studies below and standard diagnostic accuracy statistics (latent-class metrics) were calculated:

***True Positive*** (TP) correct canine alert on Xcc-positive target

***True Negative*** (TN) correct rejection, no canine alert on Xcc-negative target

***False Positive*** (FP) incorrect canine alert on Xcc-negative target, Type I error

***False Negative*** (FN) incorrect canine rejection of Xcc-positive target, Type II error

***Sensitivity*** (SEN) or true positive rate,

*SEN = TP/(TP + FN)*

***Specificity*** (SPE) or True Negative Rate

*SPE = TN/(FP + TN)*

***Precision or Positive Predictive Value*** (PPV)

*PPV = TP/(TP + FP)*

***Negative Predictive Value*** (NPV)

*NPV = TN/(TN + FN)*

***False Positive Rate*** (FPR)

*FPR = FP/(FP + TN)*

***False Negative Rate*** (FNR)

*FNR = FN/(FN + TP)*

***False Discovery Rate*** (FDR)

*FDR = FP/(TP + FP)*

***Accuracy*** (ACC)

*ACC = (TP + TN)/n*

where *n* = total number of samples assessed in each trial.

The above metrics are commonly used to evaluate the performance of diagnostic tests in medicine and other endeavors, including the evaluation of canine detectors [26,29,30,31,32]. No single performance metric can capture all aspects of canine detection accuracy. Throughout this study, we report the metrics listed above, with a particular focus on sensitivity, specificity, and accuracy. In addition to those standard metrics for test evaluation, we also examined the diagnostic capability of the canines using three graphical approaches that either depend directly on information quantities or which have direct connections to information theoretic concepts.

To illustrate the use of these methods, we focus on a single set of performance statistics derived from assessment of the impact of disease prevalence in samples on the detection performance of the canines (see Section 3.5 below). Using the summary statistics for the overall performance for canines Bady and Maxi in this set of experiments, we constructed likelihood ratio graphs [33,34], a leaf plot based on the PPV and NPV for each canine, and a “loop” plot illustrating the expected mutual information for the positive and negative diagnoses for each animal [34].

### 2.4. Detection of Xcc in Simulated New Plantings

Ruby Red Grapefruit seedlings were inoculated using a needless syringe tightly appressed to the abaxial laminar surface of 1/2 to 2/3 expanded leaves and forcing inoculum (≈10^5^ cfu/mL) into the lamina via pressure injection infiltration; then, they were allowed to develop citrus canker symptoms for 4 weeks prior to canine interrogation [35]. Non-inoculated trees were arrayed in a 100 tree, 10 × 10 grid, with ≈3 m between trees within row and between rows, and with 1 to 10 Xcc-infected trees randomly placed within the grid. Canines interrogated each tree in the grid in sequence via a serpentine pattern up and down the rows. Three detector canines associated with two collaborating commercial canine training companies assessed the randomized arrays of grapefruit trees.

**Figure 2:**
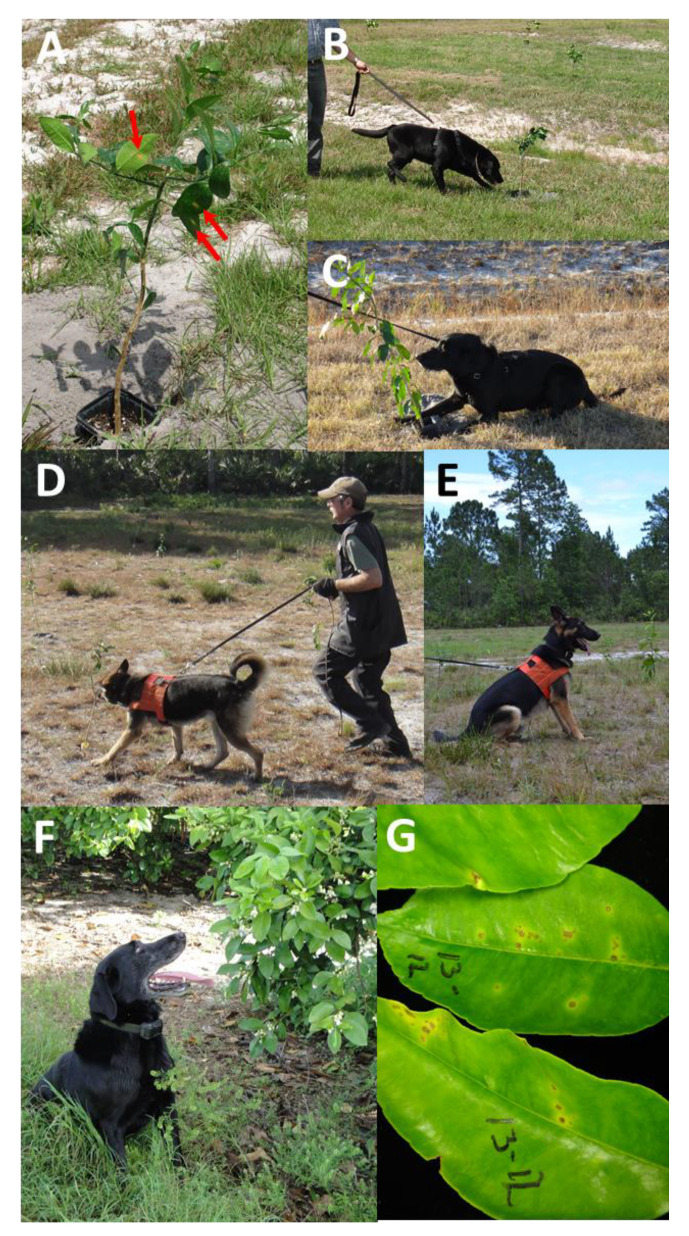
Canine detection of *Xanthomonas citri* pv. *citri* (Xcc) in simulated and commercial orchards. (**A**) Xcc-infected, potted red grapefruit inserted into ground—Xcc lesions indicated by red arrows. Detector canine “Juice”—(**B**) interrogating, and (**C**) alerting on Xcc-infected trees. Detector canine “Bady”—(**D**) interrogating, and (**E**) alerting on Xcc-infected trees. (**F**) “Juice” alerting on Xcc-infected grapefruit tree in commercial orchard. (**G**) Sample of three Xcc-infected grapefruit leaves from commercial orchard identified by “Juice”—note multiple small brown Xcc lesions surrounded by chlorotic halos.

For the first collaborating company, a single canine, a Labrador retriever “Juice”, interrogated the simulated orchard with 2, 4, 5, 7, or 10 Xcc-infected trees randomly placed within the 100-tree grid. Each of the incidence levels was replicated ten times. Tree placement was re-randomized between each replication. The experiment was repeated during four separate months, July, September, December, and May to examine the effect of seasonality. Due to commercial handler availability, not all incidence levels were examined each month (Figure 2A–C). For the second collaborating company, two canines, German shepherds, “Bady” and “Maxi”, each interrogated the simulated orchard with 1 to 6 Xcc-infected trees randomly placed within the 100-tree grid (Figure 2D,E). Tree placement was re-randomized between each replication, and care was taken to ensure that only technicians setting up each grid replicate knew the positions of Xcc-infected and non-infected trees, i.e., both handlers and canines were unaware of true positive and true negative target positions. This same “blind” test methodology was implemented through all subsequent trials. However, when a canine alerted correctly on a true positive, the technician confirmed the correct detection to the handler so the handler could appropriately reward the canine. The experiment was conducted over a two-month duration, depending upon commercial handler availability. Each canine interrogated a grid of each Xcc-incidence level at least twice. Canine alerts were recorded, and latent class metrics were calculated to assess canine performance.

### 2.5. Detection of Xcc Lesions of Various Ages

Ruby Red Grapefruit seedlings were inoculated using the methods described in Section 2.4 above. Inoculations were conducted over time, such that on the day of canine interrogation, a temporal array of Xcc-infected seedlings were 1, 3, 6, 9, and 12 weeks post-inoculation. Two canines, Bady and Maxi, each assessed a 50-tree grid (5 rows of 10 seedlings per row) five times (replications). For each replication, two seedlings with Xcc infections of the same age were randomly placed within a population of 48 additional non-infected seedlings of the same age. The experiment was repeated 7 days later using the same temporal array of seedlings, whose Xcc infections were now 1 week older. All tests were blinded as described in Section 2.4 above. Since there was no significant difference between the repeated experimental results, performance metrics were calculated for the combined data such that Xcc-infection age ranged 1–2, 3–4, 6–7, 9–10, and 12–13 weeks post-infection.

### 2.6. The Effect of Incidence of Xcc Lesions on Detection

Early observations with the canine “Juice” indicated the potential that he alerted differentially to trees infected with few versus prevalent lesions. Subsequently, canines were trained to alert to Xcc infections regardless of infection prevalence. To assess the effect of lesion incidence, Duncan grapefruit seedling trees were inoculated by pin-prick inoculation as described above to establish trees with 1, 5, 50 and 500 lesions each. Two canines, Bady and Maxi, each interrogated grids of 50 trees with one Xcc-infected tree of each lesion incidence and 46 non-infected trees. Each Xcc incidence level was interrogated twice by each canine with the location of the Xcc-infected trees re-randomized (using a random number generator) between replicates. All tests were blinded as described in Section 2.4 above. The experiment was repeated two months later with the same set of trees. Canine alerts were recorded, and latent class metrics were calculated to assess canine performance. For each lesion incidence, performance metrics were calculated considering one infected tree of the specific lesion incidence being evaluated in a population of 46 non-infected trees, ignoring the canine response of the other three Xcc-infected trees of other lesion incidence.

### 2.7. Detection of Xcc Infections in Decaying Foliage

**Figure 3:**
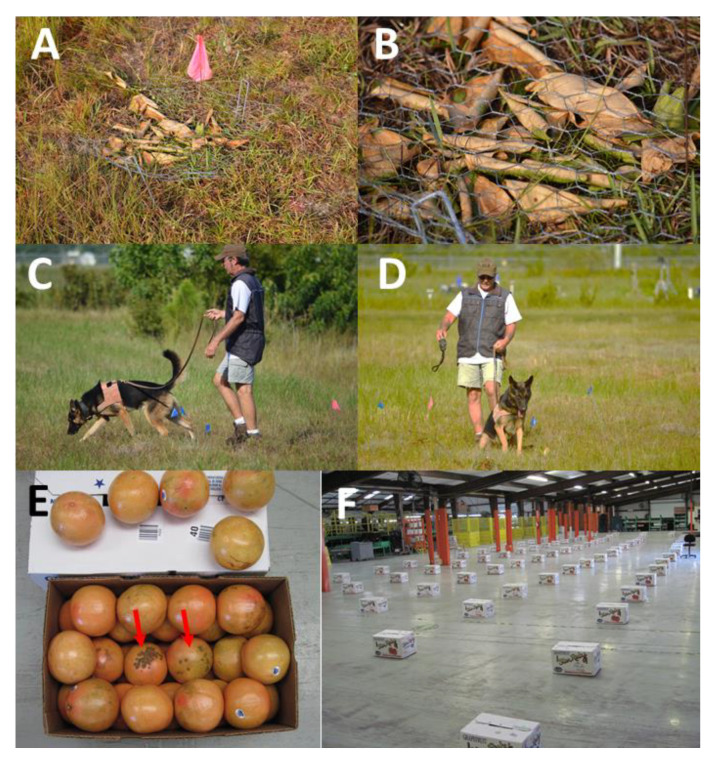
Canine detection of abscised Xcc-infected grapefruit leaves over time. (**A**) Mixed Xcc-infected and non-infected leaves in wire mesh cage, (**B**) close up of leaves in wire mesh decaying. Canine “Bady”—(**C**) interrogating, and (**D**) alerting on wire mesh cages with decaying Xcc-infected leaves. (**E**) Commercially packed grapefruit in cardboard carton with top layer of fruit removed to show Xcc-infected fruits—red arrows indicate infected fruit with Xcc lesions. (**F**) Grid of 100 cartons of commercial packed red grapefruit arrayed on packinghouse floor for canine interrogation; 1–6 cartons contain Xcc-infected fruits—positions of Xcc-infected cartons randomized between trials.

Barring adverse environmental conditions or disease, the lifespan of citrus leaves is 1–3 years prior to abscission, after which they senesce and decay. However, Xcc-infected leaves experience foliar accumulation of elevated ethylene and often abscise early. To determine the duration of canine detection of Xcc-infections in abscised leaves, Duncan grapefruit leaves, *in planta*, were infected via a needless syringe inoculation described above. Non-infected leaves were physically abscised from the trees, and 20–30 leaves were placed in wire cages as non-infected controls. True positive targets were composed of cages with 20–30 non-infected leaves, with the addition of 2–6 leaves with 30-day Xcc infections. True positive (TP) and true negative (TN) cages were randomized in an open grassy field (Figure 3A,B). Two canines, Bady and Maxi, interrogated the leaf cages on 0, 1, 2, 5, 13, and 27 days post-abscission as the leaves decayed. Each canine interrogated the leaf cages in turn and then repeated the interrogation for two to five replications on each assay date; the number of replications/day/canine depended upon weather and handler availability (Figure 3C,D). All tests were blinded as described in Section 2.4 above. Canine alerts were recorded, data were combined across replications and canines, and latent class metrics were calculated to assess canine performance.

### 2.8. Detection of Xcc in Citrus Packinghouse Environments

To examine canine performance for the detection of Xcc in a commercial packinghouse, commercially packed cardboard boxes, each containing approximately 50 red grapefruit fruits were arrayed in a 10 × 10 grid, with ≈1.5 m between rows and boxes within a row (Figure 3E,F). Within target boxes, 2 Xcc-infected fruits, each with 2–20 lesions were randomly placed in the center within each TP target carton. The target boxes containing Xcc-positive fruit were randomly arrayed on the concrete packinghouse floor and re-randomized between replications. A single canine, “Juice”, interrogated each box in the grid in sequence via a serpentine search pattern up and down the rows. The experiment was repeated twice 5 days apart. On the first day, the canine interrogated nine randomized arrays (900 cartons) with Xcc-incidence ranging from 1–4%, over a ≈3-h period, whereupon the study was halted due to excessive temperatures in an effort to preserve canine health. On the second day, the ambient conditions were more favorable, and the canine interrogated 19 randomized arrays (1900 cartons) with Xcc-incidence ranging from 1–10% over a ≈5-h period. All tests were blinded as described in Section 2.4 above. Human inefficiency of detection of packing-house inspected fruit was indicated by 100 commercial cartons selected at random post packinghouse processing, 2 of which contained 1 and 2 infected fruit with small lesions after passing multiple packing line inspections stations with trained fruit grader/inspectors and post packinghouse inspection by pathology technicians trained to detect Xcc symptoms. Canine detection of unknown true positives elucidated the human error, i.e., false negatives. These true positives were subsequently added into the grid data and considered true positives incorporated into the grid designs. Canine alerts were recorded, the results from the two days were combined, and latent class metrics were calculated to assess canine performance.

### 2.9. Assessment of Xcc Detection in Commercial Citrus Orchards

One canine, “Juice”, surveyed two commercial red grapefruit orchard blocks in Indian River County, Florida with endemic low incidence Xcc infection (Figure 2F,G). Prior to canine assessment, the blocks were surveyed visually by human assessors to determine and map the location of Xcc-infected trees. Two assessors independently examined trees visually requiring ≈5 min/tree. If a tree had unusual symptoms or was difficult to assess, one to two additional assessors examined it as well. Subsequent to canine assessment, trees on which canines alerted were visually reassessed in an attempt to determine if human assessors could confirm the canine detections. However due to the previously documented inefficiency of human visual assessment ([11,36]; T. Riley, unpublished results), it is likely that many infected trees were missed and/or could not be confirmed. Canine alerts were recorded, and the results and latent class metrics were calculated to assess canine performance against putative human visual assessment.

### 2.10. Spatial Heterogeneity of Xcc Detection Errors

It is not uncommon for detector canines to acquire a target scent at some distance from the true target, occasionally alerting on a negative target within the scent plume [37,38]. To address the concern of FN and FP canine alerts on grids of Xcc-infected and non-infected targets, respectively, we analyzed the cumulative randomized placement of Xcc-infected trees and compared it to correct Xcc true positive (TP) and true negative (TN) tree positions, calculating the distances between TP and FP and locations. We conducted this spatial assessment for (1) disease incidence of lesions in simulated orchards, (2) lesion age in simulated orchards, and (3) incidence of cartons with Xcc-infected fruit in the packinghouse.

### 2.11. Direct Detection of Xcc Bacteria

Initially, it was assumed that canines trained to detect Xcc-infected trees were alerting to a complex scent signature composed of VOCs from the bacteria plus unique plant-based VOCs produced in response to Xcc infection. We questioned if trained canines would alert directly on VOCs from Xcc bacteria without the background citrus host odor and/or unique VOCs produced by the bacteria/host interaction. To answer this question, we grew Xcc in axenic culture on nutrient agar for 1 week, harvested the bacteria, suspended them in sterile phosphate-buffered saline (PBS; 0.14 M NaCl, 1.5 mM KH_2_PO_4_, 6.5 mM Na_2_HPO_4_, 2.6 mM KCl (pH7.4)), and adjusted the suspension spectrophotometrically by diluting with sterile PBS to approximately 10^6^ cfu/mL. Subsequently, 400 µL of the suspension (containing ≈2 × 10^5^ cfu/mL) or 400 µL of sterile PBS was pipetted onto sterile cotton filter discs.

Prior to this experiment, one canine (Mi) was trained exclusively to detect Xcc *in planta* from Xcc-infected plants and a second canine (Ti) was trained to detect Xcc in vitro from axenic culture. Both canines interrogated a row of 10 metal paint cans ≈2 m apart. Into one can, a sterile cotton filter disc infused with 400 µL of Xcc dilution in PBS was placed, and into the nine remaining cans, a sterile cotton filter disc infused with 400 µL sterile PBS was placed. The canines repeatedly interrogated the line of 10 cans 10 times, the cans were re-randomized between each replication, and the study was repeated once. All tests were blinded as described in Section 2.4 above. Canine alerts were recorded, and latent class metrics were calculated to assess canine performance.

The reciprocal experiment was also conducted, wherein we questioned if canines trained exclusively on cultured bacteria could detect the bacteria *in planta*, i.e., in Xcc-infected plants. Both canines repeatedly interrogated a line of 10 trees. The line was composed of one Xcc-infected and 9 non-infected Duncan grapefruit seedlings. The plants were re-randomized between each replication, and the study was repeated once. Canine alerts were recorded and latent class metrics were calculated to assess canine performance.

### 2.12. Estimation of Bacterial Detection Threshold

Having determined that canines trained on either Xcc-infected plants or on Xcc cultures were able to detect the bacteria directly in vitro as well as *in planta*, we wanted to determine the sensitivity, i.e., lower limit of bacteria needed for canine detection. To answer this question, we grew Xcc in axenic culture on nutrient agar for 1 week, harvested the bacteria, suspended them in sterile PBS, and adjusted the suspension spectrophotometrically by diluting with sterile PBS to approximately 10^4^ cfu/mL as described above. Then, a dilution series (4 × 10^4^, 4 × 10^2^, 0 × 10^0^, 0 × 10^−1^, and 0 × 10^−2^) was prepared using sterile PBS as the diluent, and 400 µL of each bacterial dilution was pipetted onto individual sterile cotton filter discs. Immediately following the canine detection trials (described below), the individual dilutions were plated on nutrient agar, incubated for 2 days, enumerated, and the results were used to calculate the number of bacteria on the cotton filter discs (approximately 26.4, 3.60, 0.27, 0, or 0 cfu) as presented to the canines at the time of the test.

The canine (Mi) previously trained to detect Xcc-infected plants and the canine (Ti) trained to detect Xcc from culture, each interrogated rows of 10 paint cans ≈2 m apart. A sterile cotton filter disc infused with a specified dilution of Xcc in PBS was placed into one can, and into the nine remaining cans a sterile cotton filter disc infused with PBS was placed. All dilutions were interrogated by both canines and the performance was assessed. A known TP consisting of a filter pad with 400 µL of ≈4 × 10^2^ = 25.2 cfu Xcc culture was randomly placed in each line of cans as a TP control. Additionally, a TN control was assessed consisting of a single location of *Bacillus megaterium* to ensure that the canines were performing as expected. The original dilution was determined by subsequent culture to be 143 cfu/mL with ≈57 cfu/400 µL pipetted onto the sterile cotton filter disc.

To examine if canines were able to detect Xcc subcellular components, Xcc bacteria were collected from petri plate culture, suspended in PBS, and adjusted spectrophotmetrically to ≈10^2^ cfu/mL (25.2 cfu) by dilution with PBS. Then, the resulting suspension was passed through a 0.2 µm microbiological filter to remove bacterial cells and 400 µL of the filtrate was pipetted onto a sterile cotton filter disc. Filtrate was subsequently cultured and resulted in 0 cfu growth.

This “cell-free” suspension was placed in a single can and interrogated by both canines Mi and Ti in a line of 9 other cans into which were placed sterile cotton filter discs with 400 µL of PBS and one can with a sterile cotton filter disc infused with 400 µL of Xcc culture adjusted to ≈10^2^ cfu/mL = 25.2 cfu as a positive control. All tests were blinded as described in Section 2.4 above. Canine alerts were recorded and performance was assessed.

## 3. Results

### 3.1. Initial Training of Canines for Xcc Scent Signature Recognition

The Florida researchers were not involved in the initial training and evaluation of the first Xcc-detector canines in California. However, the collaborating trainer/handlers indicated that after a few days of repeated training using STU collected scent pads, the experienced criminology detector canines quickly learned and imprinted on the Xcc scent signature, alerting on scent pads from Xcc-infected trees with 95–98% accuracy (data not shown). This was a similar level of accuracy to that which trainers normally expect and achieve with criminology target scent signatures. This also led us to believe that training canines for Xcc detection was possible and thus could be viably explored further.

We established that longer sample collection times were better for recognition/training, presumably because the scent deposition on the pads was stronger, i.e., the concentration of the unique Xcc VOCs was higher. However, once trained, dogs were capable of detecting the Xcc signature on samples collected over exposure durations as low as 1 min, i.e., lower scent concentration. We also determined that the Xcc scent signature was not long lived. Once the heat-sealed, vapor-proof plastic bags were removed from refrigeration and opened to the ambient environment, the trained canines would alert on them for 2–3 weeks before the Xcc scent signature diminished to unreliable levels. Scent pad viability could be lengthened to some extent by reheat-sealing and refrigerating the pads between uses, but due to scent degradation concerns, we limited scent pad use to 2 weeks from time of collection. Therefore, the VOC composition of Xcc scent does not appear to be stable over time, unlike some scents such as human or animal scents that are very stable and can remain on clothing and other objects for long periods up to years. However, repeated experiments with a canine trained to recognize Xcc resulted in >95% correct recognition and differentiation from non-infected citrus scents, from properly handled scent pads.

### 3.2. Training Canines for Detection of Xcc-Infected Plant Material

During initial proof-of-concept studies, the 11 September 2001 terrorist attacks occurred, and many of the detector canines throughout the country were diverted to security-related tasks and became unavailable for research and our studies were curtailed. Over the next three years, Xcc had become more widely spread in Florida, reducing regulatory concerns for the movement of infected plant materials within state. Therefore, in 2004–2005, we were able to resume studies on canine detection of Xcc in collaboration with the NDDTC. However, the NDDTC’s mission is to train and deploy agricultural contraband detector canines to US ports of entry and overseas ports of departure for US travelers and freight. Our research project utilized these same canines, who upon completion of their training were shipped out to their intended assignments, as were some of the staff. Thus, we did not have consistency of canines or staff, and we rotated through canines and trainers. Even so, trainers reported >95% detection accuracy after a few days training with plants in boxes, indicating good imprinting on Xcc-infected plant materials. When deployed on a single occasion to an infected orchard, the box-trained Labrador retriever also accurately detected Xcc-infected versus non-infected leaves from the orchard. Thus, we were able to discern that canines could be trained to detect Xcc using the differential of infected versus non-infected plant materials. Due to the high turnover rate of canines and handlers, the project was again curtailed.

### 3.3. Detection of Xcc in Simulated New Plantings

Canine detection studies resumed in 2006 and continued through 2020 with three collaborative commercial canine detection companies. The performance of the first canine “Juice” indicated excellent scent signature recognition (Video S1, see Supplementary Materials). Sensitivity, specificity, precision, and accuracy ranged from 0.7333–0.9167, 0.9943–1.0, 0.8759–1.0, and 0.9733–0.9967, respectively, indicating that canine false negative (FN) alerts were slightly more prolific than false positive (FP) alerts, especially at higher incidence levels. Performance metrics indicated detection was superior for lower (<5%) Xcc-incidence, eroding slightly when incidence was ≥7%. Even so, overall accuracy was 0.9842 (Table 2).

**Figure 4:**
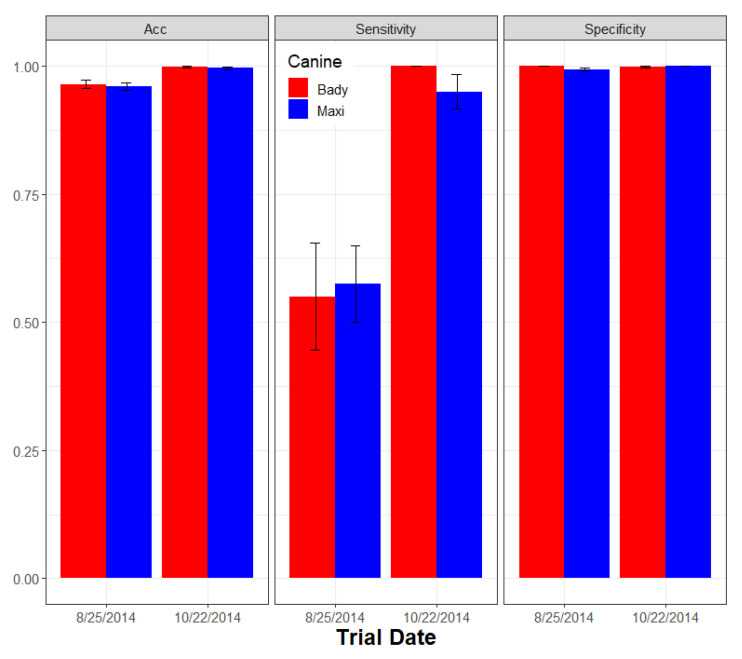
Latent class metrics for the effect of incidence of Xcc lesions on canine detection. The data demonstrate a training effect where canine detection of Xcc-infected trees (sensitivity) significantly improves between the first and second tests, which also improves slightly the overall accuracy metric. In essence, the canines learn the “game” of detecting Xcc-infected trees when presented with a grid imposed by the experimental design and become more proficient at detection over time.

Seasonality (i.e., July, summer; September, fall; December, winter; and May, spring) did not have a perceptible effect on canine detection performance when assay results were accumulated across incidence levels (Table 3). Canines were proficient at Xcc detection prior to assessing performance metrics; however, a training effect was noted. The canines improved notably in sensitivity and slightly in overall accuracy as they became more comfortable with the “game” of detection over time (Figure 4).

Since seasonality effects were not significant in the first study, performance assessment of the second two canines (Bady and Maxi) was compressed to two separate days separated by 31 days during the second study. During this second study, randomized Xcc-infected tree incidence ranged from 1 to 6% to reduce the probability of scent acquisition from nearby Xcc-infected trees when using higher incidence levels. The sensitivity, specificity, precision, and accuracy performance metrics for canine Bady ranges were 1.0–1.0, 0.9894–1.0, 0.6667–1.0 and 0.9900–1.0, respectively; whereas the same performance metrics for canine Maxi ranged slightly higher: 0.9167–1.0, 1.0–1.0, 1.0–1.0, and 0.995–1.0, respectively. The overall combined performance for the same metrics for the two canines were 0.9856, 0.9974, 0.9257, and 0.9970, respectively (Table 4). Canine Bady alerted to 11 FP and 0 FN over 2200 interrogations, whereas Maxi alerted on 0 FP and 2 FN over 2300 interrogations, suggesting that Maxi was slightly more accurate overall and both of these newer trained canines were slightly more accurate compared with Juice, the earlier trained canine. The 11 FP alerts by Bady were distributed across Xcc-infected tree incidence levels.

### 3.4. Detection of Xcc Infections of Increasing Age

**Figure 5:**
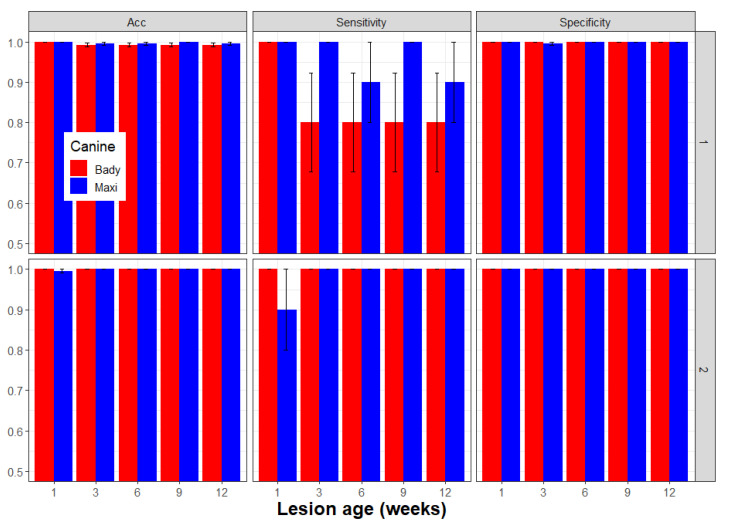
Latent class metrics for canine detection of Xcc-infections of increasing age. There was no relationship of lesion age on canine detection of Xcc-infected trees. However, the data demonstrate a training effect for canine detection of Xcc-infected trees (sensitivity) which significantly improves between the first (1) and second (2) tests as canines learn the “game” imposed by the experimental design and become more proficient at detection. Acc= Accuracy.

Latent class performance metrics for detecting Xcc lesions indicated no effect of increasing lesion age. For canine Bady sensitivity, specificity, precision = positive predicted value, and the accuracy performance ranges were 0.9–1.0, 1.0–1.0, 1.0–1.0 and 0.9960–1.0, respectively, whereas the same performance metrics for canine Maxi ranged slightly higher: 0.95–1.0, 1.0–1.0, 1.0–1.0, and 0.9980–1.0, respectively. The overall combined performance for the same metrics for the two canines were 0.9450, 1.0, 1.0, and 0.9978, respectively (Table 5). Neither canine had any FP alerts, and Bady and Maxi had eight and three FN alerts, respectively, which were distributed across Xcc-infection age groups with no apparent effect of age (Figure 5).

### 3.5. The Effect of Incidence of Xcc Lesions on Detection

**Figure 6:**
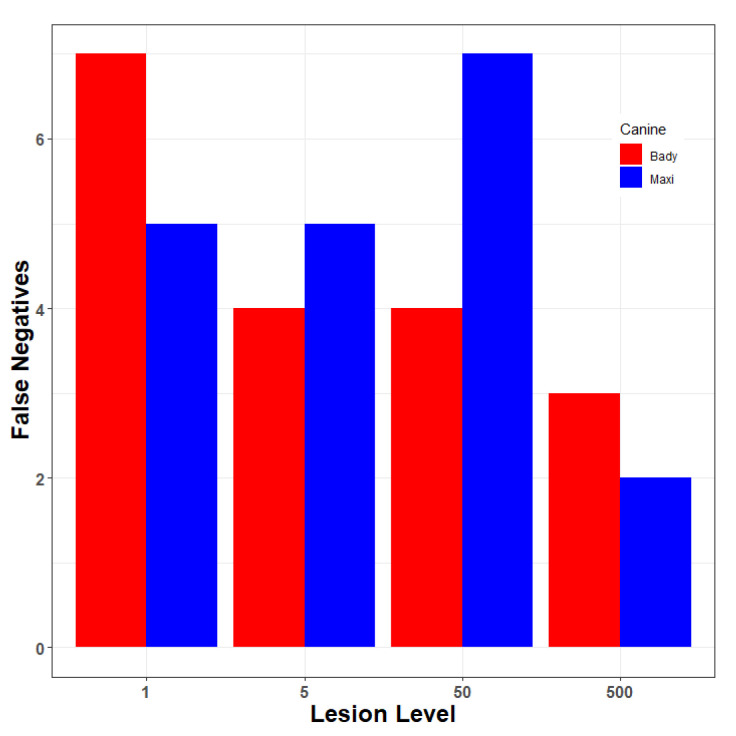
Effect of lesion incidence on false negative canine detections. The data demonstrate a general erosion of canine detection of Xcc-infected trees (sensitivity) as the incidence of infection within individual trees increases. As the scent signature becomes stronger due to heavy infection in some trees, canines begin to false alert on nearby trees because they acquire the scent farther away from the true source.

Across all lesion incidence levels, both canines had a higher prevalence of FN than FP alerts (FDR = 0.1159, FPR = 0.0022, overall). Canine Bady had fewer FP than Maxi, but this effect was not consistent across the range of lesion incidence (Figure 6). The sensitivity of both canines increased with lesion incidence (0.6750 to 0.8750), whereas specificity remained high (0.9978) as did overall accuracy (0.9910–0.9952) across all incidence levels (Table 6).

**Figure 7:**
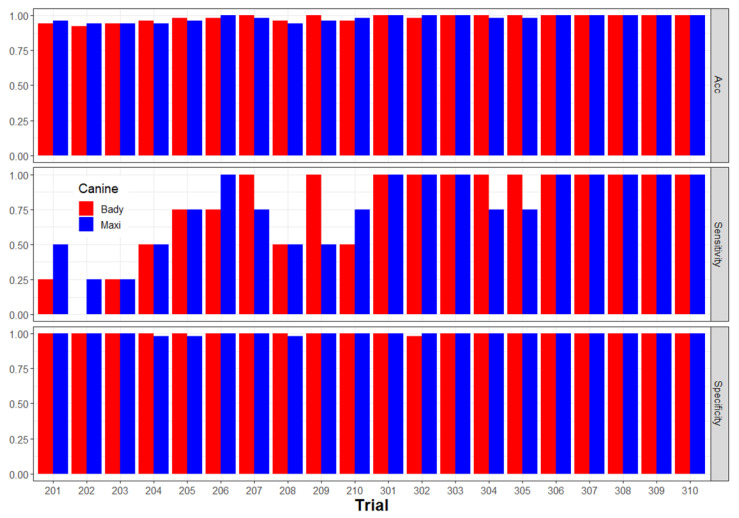
Effect of Xcc lesion incidence on overall accuracy, sensitivity, and specificity of canine detections. The data demonstrate a general improvement in the sensitivity of canine detection as training experience for both canines was accumulated over an increasing number of trials, whereas overall accuracy was high throughout and improved only slightly over accumulated trials and specificity remained high and stable. We use the overall results for each animal displayed in Table 6 to illustrate the diagnostic performance of the canines Bady and Maxi in an information theoretic framework. Figure 8 shows the results of this exercise.

Both PPV (0.8710–0.8974) and NPV (0.9930–0.9973) remained relatively constant across Xcc incidence, indicating that canines were superior in predicting actual Xcc-non-infected trees (NPV) and slightly less predictive of actual Xcc-infected trees (PPV), although both canines had low false positive rates (FPR). These data also demonstrated the advantage of repetitive training. This training effect was seen as a general improvement in the sensitivity of Xcc detection with accumulated experience over an increasing number of trials. Conversely, accuracy remained high and improved only slightly, and specificity remained high and stable throughout (Figure 7).

The sequence of plots illustrates the diagnostic performance of the canine starting with the graphical summary provided by the likelihood ratio plot, which summarizes diagnostic performance using metrics that are independent of disease prevalence. The positive likelihood ratio (LR+ = TPP/FPP) is the slope of the solid line segment: for each animal, the values are Bady = 701.5, Maxi = 530.4. The slope of the dashed line segment is the negative likelihood ratio for each animal (LR− = 1 − TPP/(1 − FPP) = FNP/TNP). The values for each animal are Bady = 0.24, Maxi = 0.13. Generally, for effective diagnostic performance, LR+ >> 1 and LR− << 1 are required. Both canines achieved effective positive and negative diagnostic performance, with positive performance (i.e., confirmation of pathogen presence) being particularly effective.

**Figure 8:**
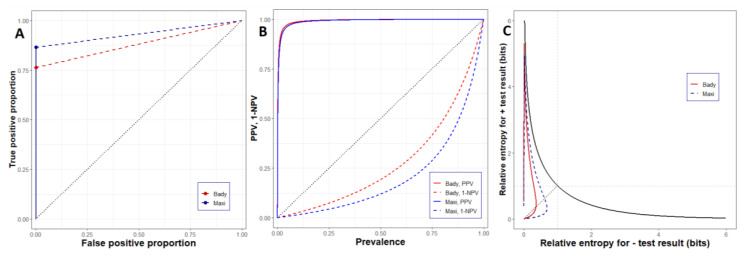
(**A**) Likelihood ratio plot for canines Bady and Maxi, based on average performance over a range of pathogen prevalence values in citrus canker detection trials. The axes are the same as those for a receiver operator characteristic (ROC) curve, each canine being represented by a single point. In general, the closer the point for the animals to the upper left corner with TPP = 1, FPP = 0, the better the overall diagnostic performance. The gradient of the solid line section is the positive likelihood ratio for cases for each canine. The gradient of the dashed section is the negative likelihood ratio. (**B**) A predictive value leaf plot for Bady and Maxi based on the likelihood ratio values displayed panel (**A**). The plot displays the relationship between possible disease prevalence (or prior in a Bayesian framework) and the possible post-diagnostic probability of disease given either positive or negative diagnostic outcomes. The canines have very similar positive alert performance, but they differ in the information they provide in negative alerts. In general, a negative alert by Maxi provides more information than one by Bady. For both canines, positive alerts result in a high post-test probability of disease even at low prior disease values. (**C**) A relative entropy “loop” plot for each animal based on the same likelihood ratios. For each animal, disease prevalence increases clockwise around the loop which shows the expected information supplied (in bits) for a positive vs. negative alert at each possible disease prevalence between 0 and 1 in steps of 0.0001. In effect, the loop plot shows the information gain from alerts corresponding to the change in probable disease prevalence following diagnosis displayed in the leaf plot in panel (**B**).

Likelihood ratios derived from TPP, FPP, TNP, and FNP allow Bayesian updating of disease prevalence to give disease prevalence conditional on diagnostic outcomes; i.e., they can be used to produce predictive values. Figure 8B shows a leaf plot for Bady and Maxi, based on the likelihood ratios displayed in Figure 8A. The leaf plot shows values for PPV and (1-NPV) as functions of initial disease prevalence over the range of prevalence (0,1). To interpret the plot, select a value for prior Xcc prevalence. Locate the value on the diagonal of the plot. To read off the post-diagnostic probability of Xcc presence following either positive or negative diagnosis, trace up or down (respectively) from the point on the diagonal until intersecting with the curves for either canine. The PPV and 1-NPV values can be read off the vertical scale from reading across from the upper and lower curves (respectively); the 1-NPV value being converted to NPV by simple arithmetic thereafter. We draw attention to the high posterior (i.e., PPV) probability of Xcc presence generated by both Bady and Maxi even at low prior disease prevalence. Figure 8B also illustrates that, in general, Bady and Maxi provided more information in positive than negative diagnostic outcomes.

Following Hughes et al. [34], we calculated the expected mutual information (i.e., relative entropy) for positive and negative diagnostic outcomes (I+ and I−, respectively) for Bady and Maxi corresponding to the likelihood ratios in Figure 8A and the predictive values in Figure 8B. The resulting “loop” plot is shown in Figure 8C. The black curve, descending from left to right across the plot, shows the theoretical maximum information (in bits) that could be obtained from an error-free definitive diagnosis of disease status for an unknown sample. Disease prevalence increases from 0 to 1 sequentially along the curve from left to right, indicating that at low disease prevalence, a positive diagnostic outcome contains more information than a negative one, with the opposite being true when disease prevalence is high. The short diagonal line intersecting the curve shows values where I+ = I−. Note that this diagonal intersects the curve at a value of I+ = I− = 1 bit, which occurs at a disease prevalence of 0.5; in effect, this corresponds to seeing the result of a coin flip.

Corresponding to the results in Figure 8B, the information loop plot shows clearly that Bady and Maxi provided more information about Xcc disease status in positive than in negative diagnostic outcomes. The maximum information supplied in a positive diagnosis for either canine was in the order of 4.5 bits, whereas the maximum value for negative diagnoses was less than 1 bit.

### 3.6. Detection of Xcc Infections in Decaying Foliage

On the day the leaves were abscised from infected trees (day 0) canines correctly identified all positive and negative leaf piles without error. There were very few FP errors throughout the study (3/448). However, as time post-abscission increased FN errors increased from 1 to 20 for days 1 and 27 post-abscission, respectively. On day 27, neither canine detected any of the decaying 20 Xcc-infected leaf piles after repeated attempts, i.e., FN error = 100% (Table 7).

### 3.7. Detection of Xcc in Citrus Packinghouse Environments

**Figure 9:**
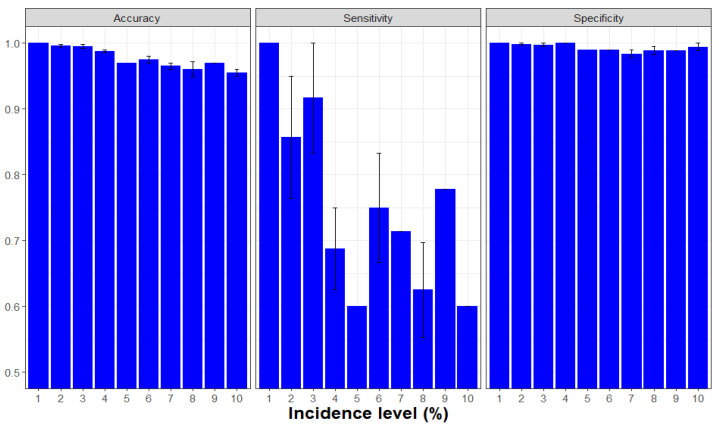
Effect of incidence (proportion) of commercial fruit boxes containing Xcc-infected grapefruit on false negative canine detections. The data demonstrate a general erosion of canine detection of Xcc-positive boxes (sensitivity) as the incidence of infected boxes increases within the grid in the packinghouse. As the scent signature becomes more prevalent within the test grid commensurate with the number of boxes containing Xcc-infected fruit, canines begin to false alert on nearby boxes in close proximity to boxes with actual infected fruit because the canines acquire the scent farther away for the true source.

Canine Juice detected just a few Xcc lesions on 1–2 grapefruit fruits when commercially packed into cartons of ≈50 fruit per carton, especially at low incidence. FN errors generally exceeded FP errors throughout the packinghouse study. However, the total number of FN + FP errors began to increase when ≥4% Xcc-incidence of cartons were arrayed in the grid of 100 cartons on the packinghouse floor (Table 8, Figure 9 and Video S2, see Supplementary Materials).

We conducted higher Xcc-incidence interrogations later in the day, which corresponded to a general rise in temperature in the packinghouse environment, resulting in fatigue of the canine. It was observed that the canine often acquired the Xcc scent signature from cartons farther away depending on the direction of the airflow. This was evidenced by the canine’s desire to disperse with the required serpentine search pattern through the grid in favor of going directly to a detected target at some distance. Additionally, the spatial proximity to other Xcc-infected cartons as well as the prior locations of Xcc-infected cartons with residual odor led to some FP results (see spatial analyses below).

### 3.8. Assessment of Xcc Detection in Commercial Citrus Orchards

**Figure 10:**
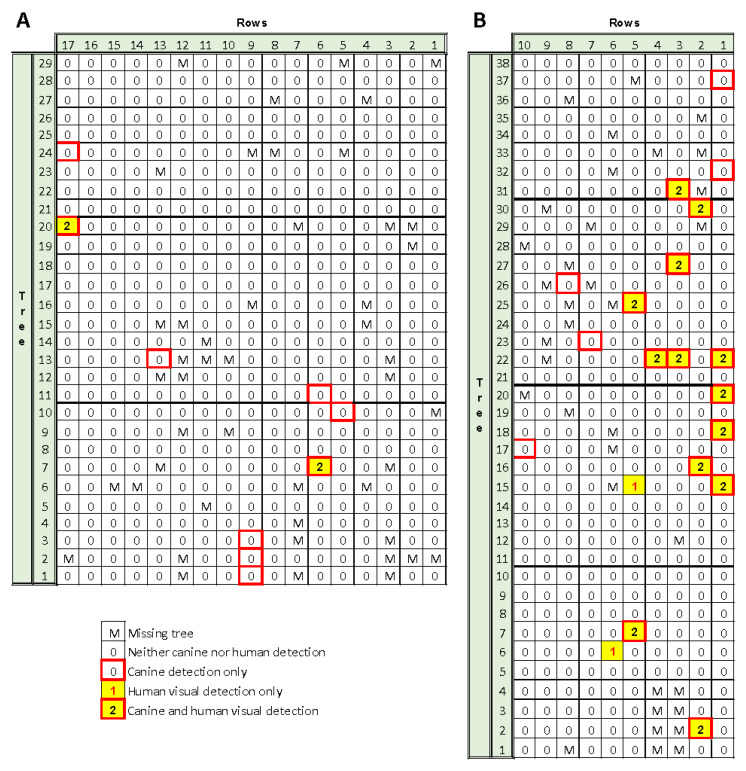
Canine versus human visual detection of Xcc-infection in commercial citrus orchards in Indian River County, Florida. (**A**) Orchard 1—Mature 42-year-old red grapefruit on sour orange rootstock, (**B**) Orchard 2—7-year-old red grapefruit.

For the canine Juice, sensitivity, specificity, precision = positive predicted value, negative predictive value and accuracy performance metrics were 1.0, 0.9842, 0.2222, 1.0, and 0.9843 for Orchard 1, and 0.8667, 0.9848, 0.7222, 0.9939 and 0.9797 for Orchard 2, respectively (Table 9). When we progress from a known infection status of potted trees in a simulated orchard (composed of confirmed Xcc-infected and non-infected trees, held in an isolation greenhouse to ensure no additional infections — see simulated new planting results above) to a commercial orchard (Figure 10) (in which disease status is unknown and subject to human visual confirmation and errors), detection precision metrics declined. This decline relates to our inability to determine the infection status unequivocally via human visual inspection. As previously demonstrated, in mature trees it is difficult-to-impossible to detect all incipient and low incidence infections by human visual inspection ([11,39]; T. Riley, unpublished results).

### 3.9. Analyses for Spatial Heterogeneity of Xcc Detection Errors

**Figure 11:**
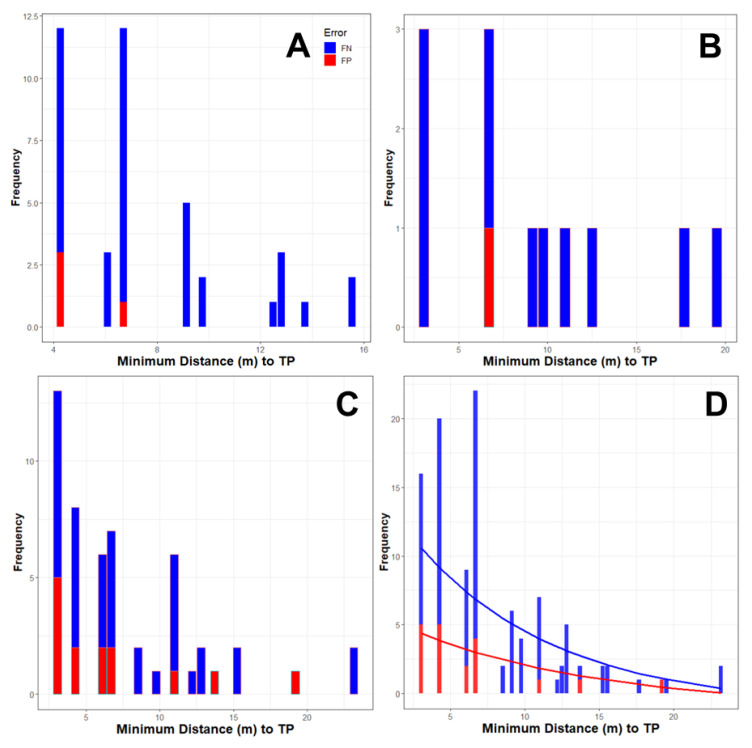
Spatial heterogeneity analysis of canine detection errors for trials of (**A**) lesion incidence, (**B**) lesion age, (**C**) packinghouse, and (**D**) combined data A through C. In all three trials there was a greater number of false negative (FN) than false positive (FP) errors. All trials had a greater prevalence of FN errors and errors were more prevalent at shorter distances from a true positive (TP). Distance is presented as multiples of the distance between plants or cartons (packinghouse) in the grid, i.e., 3.048 m (10 ft) within and between rows.

Sufficient data were available for the spatial analysis of detection errors for three portions of the overall Xcc detection study for the following trials: (1) Disease incidence of lesions in simulated orchards, (2) Lesion age in simulated orchards, and (3) Incidence of cartons with Xcc-infected fruit in the packinghouse. In all three trials, there were more FN than FP errors. Errors were omnidirectional and more prevalent at shorter distances, especially <6.5 m from a true positive (TP) (Figure 11).

### 3.10. Direct Detection of Xcc Bacteria

The canine Mi trained on *in planta* Xcc-infected trees was competent at detecting Xcc bacteria harvested from in vitro axenic cultures (Videos S3 and S4, see Supplementary Materials). The reciprocal was also true, in that the canine Ti trained to detect Xcc from in vitro axenic cultures was able to detect the pathogen *in planta* from Xcc-infected trees (Videos S5 and S6, see Supplementary Materials). Both canines detected the reciprocal target without any additional training. There was a slight trend in that canine Mi was slightly superior at detecting Xcc-infected plants and canine Ti was slightly better at detecting Xcc from culture (Table 10).

### 3.11. Estimation of Bacterial Detection Threshold

The result that canines trained on either Xcc-infected plants or on Xcc axenic cultures can detect the bacteria directly in vitro as well as *in planta*, with near equivalent accuracy, implied that canines may be detecting the bacteria directly in plants and not volatiles generated by the host plants in response to infection. Therefore, we sought to determine the detection limits, i.e., the lowest concentration of bacteria required by the canines for scent detection. Thus, both canines were challenged with the task of detecting Xcc harvested from axenic culture, suspended in PBS, over a range of dilutions from ≈4 × 10^2^ to 0 × 10^−2^ cfu as described above, with final bacteria on sterile cotton filter discs estimated to be 26.4, 3.60, 0.27, 0, and 0 cfu. Both canines detected Xcc throughout the dilution range, indicating that a sufficient concentration of the Xcc scent signature was present throughout the range tested (Table 11 and Videos S7 and S8, see Supplementary Materials). Neither canine reacted to the *B. megaterium* isolate at the estimated 57 cfu on the scent pad, indicating that the scent signature was apparently specific to Xcc bacteria and not a ubiquitous bacteria scent. Neither canine reacted to a 1 × 10^2^ cfu/mL concentration of 0.2 µm bacterial culture filtrate, indicating that the subcellular component(s) composing the scent signature was not diluted out at any concentration tested but instead was filtered out. This indicates that the canines are detecting Xcc at below the cellular population level, i.e., below a single bacterial cell.

## 4. Discussion

### 4.1. Canine Detection of Xcc in Simulated Plantings and Information Theoretic Analyses

Through the multiple trials of this study, we were able to demonstrate unequivocally that canines can discriminate and detect Xcc *in planta* (in infected foliage and fruit) by the use of canine olfactory surveillance in simulated and commercial orchards and packinghouses.

We found that canines alerted to Xcc infections over a range of lesion ages from 1 to 12 weeks post-infection with equal accuracy. These results indicate that the scent signature of Xcc-infection as perceived by canine detectors does not change significantly, if at all, with age of infection. At one week post-infection, the lesions are not yet erumpent and only visible with 10× magnification. At the non-erumpent stage, the numbers of bacteria in such lesions are low and do not directly expose the bacteria to the environment (i.e., lesions have not broken through the intact epidermis and cuticle), yet a sufficient concentration of scent signature appears to emanate from leaves for canine recognition.

Canines were tested in simulated plantings with 2% to 10% (canine Juice initial test) and 1% to 6% (canines Bady and Maxi) incidence of infected trees. In the initial test with the canine Juice, sensitivity increased with lesion incidence, whereas the specificity and overall accuracy remained static across all incidence levels. In a more expansive test with two more extensively trained canines, sensitivity for canine Bady appeared to be unaffected by incidence of infected trees, whereas the sensitivity of canine Maxi seemed to erode slightly with increased incidence of infected trees. Bady had a number of FN alerts, eroding specificity and precision somewhat that were not related to infected tree incidence, whereas Maxi had none and experienced no such erosion. Overall, canines were superior at predicting Xcc-non-infected trees (NPV) and slightly less predictive of actual Xcc-infected trees (PPV). As a field deployable early detection tool, a slight trend toward false negatives is usually accepted by growers, as they are willing to tolerate an assay that misses a few infections rather than misidentifying disease-free trees as diseased, resulting in tree removal and commensurate loss of production.

Xcc lesion populations can range from a single lesion to thousands per tree depending on inoculum prevalence and the susceptibility of tissues following an inoculum dispersal event. Across the range of lesion incidence levels assayed, both canines had a higher prevalence of FN than FP alerts. The sensitivity of both canines increased with lesion incidence, whereas the specificity and overall accuracy was >99% across all incidence levels with low false positive rates (FPR).

Information theoretic analyses of the overall diagnostic performance of both canines showed very clearly that the information provided by positive diagnostic outcomes was far in excess of that provided by negative outcomes, which is in relation to the impact on the probable presence of Xcc. We used a graphical approach recently developed by Hughes et al. [34] to illustrate the diagnostic capacity of the animals. One noteworthy aspect of canine performance is their very high positive likelihood ratios for cases. It has been noted by several authors that disease diagnosis as a tool in decision making at low disease prevalence is problematic [40,41] because, with the diagnostic capability of many commonly used approaches, the post-test probability of disease is still relatively low, even after a positive test outcome, and the sampling error is pervasive (see Section 4.5 below). The positive likelihood ratios achieved by the canines assessed here are in the region of two orders of magnitude higher than values reported in the literature for many plant and human diseases [38,42,43]. As illustrated in Figure 8B this diagnostic capacity allows for effective disease screening even when the background disease prevalence is low.

### 4.2. Detection of Xcc Infections in Decaying Foliage

Lesions of Xcc cause the surrounding citrus tissues to produce ethylene, which can cause abscission if lesions occur near the junction where a leaf petiole joins a stem. When Xcc-infected leaves fall to the ground near citrus trees, rain or irrigation splash can cause inoculum dispersal and re-infection. However, Xcc survival in lesions of decaying leaves decreases exponentially over time [44,45]. Canines were able to detect Xcc in lesions when leaves were newly abscised, but canine detection eroded over time. As time post-abscission increased, FN errors increased, and canines were unable to detect Xcc in leaves 27 days post-abscission. The results indicate a rapid decline in the emanation of Xcc scent signature over time as Xcc-infected foliage decays live Xcc population concurrently decline, resulting in an increase in FN errors through time post-abscission and that abscised infected leaves may not be a reliable indicator of a tree’s infection status.

### 4.3. Detection of Xcc in Citrus Packinghouse Environments

Canine detection of Xcc lesions in boxed citrus fruit in the packinghouse was exceptional considering the noisy, hot, and highly distracting environment. Total FN + FP errors increased when the incidence of Xcc-incidence of cartons was ≥4%. We conducted higher Xcc-incidence interrogations later in the day, which corresponded to a general rise in temperature in the packinghouse environment, causing fatigue of the canine. Additionally, the spatial proximity to adjacent or proximal Xcc-infected cartons may have led to FP alerts. Furthermore, FP alerts occasionally occurred due to persistent residual odor when Xcc-free cartons were relocated (during re-randomization between replications) to where a prior Xcc-infected carton had previously resided. This issue has been noted often by canine trainers across an array of target odor types. Therefore, we re-examined the data for error effects due to spatial proximity to Xcc-infected cartons and residual scent signature odor temporarily permeating the concrete packinghouse floor (see Section 4.4 below).

In an initial study prior to the full experiment, we noted that the canine alerted on two cartons that had recently passed through the packing line. These cartons were visually inspected thoroughly at multiple points in the packing line by commercial packinghouse inspectors and were determined to be Xcc-negative. At first, we thought these were FP detections, but when the grid was re-randomized and the cartons were relocated to different positions, the canine continued to alert on these same two cartons. Upon careful re-inspection, we determined that these two cartons contained a single fruit each with one and two small lesions (≈1 mm dia.), respectively. These observations demonstrate the keen sensitivity of canines to detect even a trace amount of odor and that human visual inspection has limitations for the detection of low-incidence infections especially with small lesions. Canine detection in the packinghouse is superior at low incidence levels. Current detection and elimination/discard of infected fruit were conducted by multiple skilled inspectors observing fruit as it passes through the multiple designated visual examination stations of the packing line. As expected, small Xcc lesions are the most difficult for inspectors to detect and differentiate from other small blemishes. However, Xcc lesions that go undetected can cause a rejection of fruit shipments when inspected at ports of destination. Thus, a highly sensitive detection tool such as canines that does not depend on visual detection could greatly enhance the sanitation of packed fruit shipments and diminish the proportion of rejections at destination markets.

### 4.4. Spatial Heterogeneity of Xcc Detection Errors

A spatial assessment of the detection data allowed us to determine if the proximity of Xcc-positive trees positioned immediately adjacent to, at oblique angles to, or upwind from canine FNs or FPs were related to the cause of the error. In the trials of this study, the majority of errors, both FN and FP, were within 1 to 2 plant or carton (packinghouse) spaces (within row, across row, or at oblique angles) of a TP. Errors also became more prevalent when the incidence of TP targets increased within the trial grids. However, there was no prominent directionality to errors. False positive alerts can be caused by the dissemination of the odor plume around an odor source. Jezierski states, “*Ideally, dogs should alert as close as possible to the site where the odorous material is hidden by comparing the differences in odor concentration inside the odor plume. It is common for a dog to enter, then exit and reenter the scent cone during odor detection, which may account for the number of times a canine passed a hide as demonstrated in the data. The role of the distribution of the odor plume was evident in our experiment when comparing the percentage of false alerts in particular searching sites. When searching outdoors, the distribution of the odor plume may often enable a more easily directional scenting and localization of odor source, which thus takes less time with more correct and fewer false alerts*” [46]. Craven found that the odor plume of a drug moves and disperses, depending on air currents, humidity, temperature, or features in the terrain, which may also influence the detection performance [47]. The fluid dynamics of odor transport during canine scent detection is highly complex and has not been examined extensively. Angle stated that, *“Much more research needs to be conducted in order to understand the movement of biological VOC within the thermal plume (e.g., microcurrents) and in the aerodynamic wake/wind currents in order to develop search patterns to optimize biomedical detection”* [39].

Although errors were relatively few overall, FN alerts were more common with Xcc detector canines than FP alerts. In an orchard environment, airflow is highly channeled between hedge rows of trees, dynamic, and convoluted with ubiquitous eddies. The complexity of using canines as detectors in a highly heterogeneous open-air commercial orchard environment, or indoor in a harsh noise and distraction rich packinghouse, presents a myriad of potential causes for error, not all of which do we understand or recognize. Our data indicate that the relatively few FN errors increases slightly with an increase in the incidence of TP targets in the environment, especially when these TP targets are in close proximity. One explanation could be that when a canine correctly locates a TP (TP1), infrequently, it can be confused by another TP (TP2) in close proximity. In this example, the canine may misinterpret that the scent it acquired near TP2 is originating from TP1, disregarding TP2 as an additional scent source. Conversely, if the scent emanating from TP1 is of significantly greater concentration than the scent emanating from TP2, the canine might track to the “maximum scent concentration” of TP1, thus ignoring the “minor” scent signature of TP2.

It is well demonstrated that canine detectors can pick up a target scent at some distance. In our experience, training canines for Xcc detection, and additionally for CLas [26], plum pox virus, and vegetable virus detection (unpublished), we have often observed that canines acquire a scent signature at considerable distance from the known target. This is consistently experienced when training canines in spatial grids of predominantly non-infected plants with a low incidence of pathogen-infected plants randomly placed in the grid. Normally, we urge the canine to interrogate each plant in the grid in a serpentine pattern up and down each row so that we can ensure that all plants are assessed equally and collect performance data. However, if the canine is allowed to interrogate the grid off leash, it will often acquire a target scent plume and divert its trajectory obliquely across multiple rows directly to an infected plant: then, it will alert. In other words, the canine has already acquired a viable target at a considerable distance, and it is likely that by odor strength and gradient characteristics, the canine developed a mental picture of the target’s estimated location that the canine refines as it hones in on the source.

### 4.5. Assessment of Xcc Detection in Commercial Citrus Orchards

As intimated in the results above, when we examined canine performance in grids for simulated orchards, lesion age, and lesion incidence, we had unequivocal knowledge of the Xcc infection status of all individual trees. This is because we performed the inoculations, enumerated the Xcc infections on each tree, and all trees were maintained in an isolation greenhouse prior to use where disease spread was highly unlikely. However, in a commercial orchard environment, precise mapping of disease is immensely more difficult. In orchards, we were hindered by the difficulty of Xcc detection and our reliance on human visual survey, especially when trying to accurately map low incidence infections. Tree canopies can be large, composed of >100,000 leaves per tree, and some leaves are in difficult-to-view positions — high in a tree or visually occluded by surrounding branches and foliage. Angle and quality of light, cloudy or bright sun can enhance or diminish visual acuity. For example, when visually surveying trees during the citrus canker epidemic for Xcc infections during the Citrus Canker Eradication Campaign in Miami, Florida, it required an average of 106 days (range 30–270 da) post-infection to visually detect Xcc infections in trees on residential properties. Thus, incipient and low incidence infections were rarely found [11,48]. In a study conducted by USDA, APHIS to determine the efficacy of visual detection of Xcc, 18 two-person teams of trained inspectors surveyed a 12.1 ha (30 ac) block of commercial citrus with known Xcc infections. No two teams detected the same Xcc-infected trees, no teams detected all Xcc-infected trees, and each team found Xcc infections and infected trees previously unrecorded. [Riley, unpublished results].

In the current study, in Orchard 2, the canine alerted on five trees previously unconfirmed by human visual assessment (Figure 10). In an attempt to confirm or refute the canine alert on one of these trees, two highly trained technicians required over two hours on the ground and climbing in the 7-year-old tree, ≈3 m tall canopy to find a single leaf lesion that was obscured by sooty mold. Other lesions may have existed in this tree as well but eluded visual inspection. Visual inspection was limited due to the large number of trees that needed to be visually scrutinized for low incidence lesions and because of the immense amount of time needed to fully assess each tree. Therefore, the infection statuses of these five trees were categorized as FP alerts by the canine (Table 9, Orchard 2). However, if we give the benefit of the doubt to the canine and assume that these trees escaped human visual detection, and were thus TP trees that were correctly identified by the canine, the precision metrics improve considerably, with sensitivity, specificity, precision, negative predictive value, and accuracy performance metrics rising to 0.9, 1.0, 1.0, 0.9939, and 0.9942 (Table 9, Orchard 2—Theoretical). Thus, the precision = positive predicted value (PPV) increases from 0.7222 to 1.0 and overall accuracy increases from 0.9797 to 0.9942, both theoretically.

Humans have great difficulty detecting Xcc in tree canopies with low Xcc-infected leaf incidence. Whereas the canine was a highly sensitive detector (i.e., discovered one lesion within a large canopy) that very rapidly detected the infection and alerted within 1–2 s. The differential between canine and human sensitivity and speed, canine-2 s vs. human team-2 h, becomes apparent and exemplifies the significant differences in probability of detection. These differences translate directly into cost of survey, leaning heavily toward the superiority of canine detection. Additionally, from our experience, it was inappropriate to use a less sensitive detection method (human visual) to validate a more sensitive method (canines). This is demonstrated by the example of commercial Orchard 2 (Table 9, Figure 10), for which five TN trees (as determined by human visual survey) were probably incorrect although identified by the trained canine detector as alerts, and therefore diminished the true estimates of canine detection performance.

### 4.6. PPV Versus NPV, the Choice between Risk-Aversion and Risk-Acceptance

Growers and regulatory agencies need to ask themselves what is more important depending upon their detection requirements. Is it important to detect all Xcc-positive plants (PPV = 1.0), even if it means a few FP plants (NPV < 1.0) will be indicated as well (i.e., risk averse: willing to cull or treat some Xcc-negative plants in an attempt to best control/mitigate a disease epidemic)? Conversely, does a grower prefer a diagnostic that never falsely implicates a plant as Xcc-positive (NPV = 1.0), and is willing to accept a few FN indications (PPV < 1.0) (i.e., risk accepting: does not want to cull or treat any non-Xcc-positive plants, for example attempting to avoid an adversely harsh regulatory response during an eradication campaign)? Ideally, we want a diagnostic with the highest PPV and NPV possible. However, PPV and NPV are influenced by disease prevalence [30,31]. For example, if we hold sensitivity and specificity constant, the lower the disease prevalence, the higher the PPV. In contrast, as the incidence of the disease increases in a population, PPV improves. Therefore, when evaluating the PPV and NPV metrics for canines or any other diagnostics, we need to consider both the disease incidence within the population and the population size tested.

In general, near-perfect diagnostics are rare in both medicine and plant pathology for a wide array of reasons. Even near-perfect diagnostics are often plagued by sampling error. For example, PCR detection of another citrus bacterial pathogen, CLas, the causal agent of citrus Huanglongbing, is near 100% accurate when testing infected tissue, but when used to assay field trees, it often gives FN results with accuracy of ≈20% due to sampling error, due to the scarcity of infected cells even in systemically infected trees [26]. In mature trees with >100,000 leaves, selecting a leaf with CLas even from a systemically infected tree or even selecting tissue with CLas from an incompletely infected leaf can be very improbable. In contrast, the canines are interrogating the tree holistically; that is, the CLas scent signature can be acquired regardless of where the bacteria are located in the tree, which circumvents both the potential paucity of CLas-infected tissue and the sampling problem. Canine detection of Xcc exhibited many of these same CLas hallmarks, although Xcc and CLas are very different types of phytobacteria. As noted above, in one case, the canine detected Xcc in a 7-year-old field tree with a single lesion obscured by sooty mold that required two trained technicians over 2 h to locate, and in another case, the canine detected single small lesions in fruit cartons missed by trained inspectors. Whereas human inspectors must spend several minutes examining each tree, canines trot along a row of orchard trees interrogating at a rate of ≈1 tree/2 s, continuously drawing in air parcels multiple times per second, thus efficiently surveilling large orchard areas quickly. Molecular or serological detection methods require collecting multiple samples per tree, returning to the lab to process and assay, and the use of moderate to extensive consumables; in addition, assay results are delayed depending upon laboratory backlog and are expensive (sampling and assay costs can exceed $40 US per sample at the time of this writing). Conversely, canines are rapid (1–2 s/tree), results are essentially instantaneous, more cost effective (≈$4.50/tree depending on orchard size, conditions, access, etc.), and more accurate due to the lack of sampling issues when using canines versus other detection methodologies [26].

Additionally, Xcc detector canines were successfully deployed by a commercial canine detection company to assess a commercial citrus nursery and successfully detected multiple Xcc-infected nursery plants unknown to the nurseryman, although no quantification of detection was documented. Such early detection can ensure that Xcc-infected plants are identified, eliminated in the nursery, and thus not transplanted to orchards. Avoiding the introduction of initial inoculum when establishing new plantings is an obvious advantage to mitigating an otherwise potential epidemic. Our collective results from this study imply that canines can be trained as viable early detectors of the pathogen in various agricultural environments, including citrus orchards, packinghouses, and nurseries.

### 4.7. Direct Detection of Xcc Bacteria and Estimation of Bacterial Detection Threshold

We also trained canines exclusively to two targets: Xcc-infected trees and Xcc axenic cultures. We found that canines trained to either target inherently detected the heterologous targets, although both canines were superior at detecting the homologous target they were trained upon as opposed to the heterologous target. This implies that the scent signature does not need to be augmented with background citrus host VOCs and/or unique VOCs produced by the bacteria/host interaction. Therefore, Xcc cultures might be a sufficient training target if Xcc-infected plants are not available such as in a quarantine situation. In a recent study of five and four respiratory viruses and bacteria, respectively, researchers detected 12 and six VOC that were associated with bacterial and viral growth, respectively, and they identified two VOCs that differentiated bacterial and viral infection [49]. Angle and colleagues discuss the opportunity and complexity of discriminating VOC biomarker detection from diseased individuals [50]. Thus, the use of canines to detect and discriminate a phytobacterial pathogen is not unfounded. The precise and discriminating biomarker VOCs detected from Xcc bacterial cultures needs further examination. Such examination should include multiple canines and perhaps determination of the optimum Xcc-culture concentration on which to train canines to achieve optimized Xcc-infected plant detection, which is beyond the scope of this study.

In a deeper examination of direct in vitro detection, canines trained to either target were able to detect highly dilute Xcc culture solutions as low as 0 × 10^−2^ cfu, i.e., 100-fold less than a single bacterial cell. This implies that the scent signature is composed of bacterial cell VOC constituents or exudates that occur at concentrations many-fold that of the bacterial cells. To explore this further, we had the canines interrogate a bacterial culture filtrate and found that the canines did not react to the filtrate without bacterial cells present. When considered concomitantly with the canines reacting to culture dilutions as low as 10^−2^ cfu/mL, this led us to suspect that the canines are reacting to a subcellular component that is larger than the 0.2 µm filter pore size. Axenic bacterial cultures are composed of both intact cells and fragments of older dead and decomposing cells. One possibility is that bacterial cell fragments may play a role in the Xcc scent signature. The chemical fractionation and canine testing of such of filtrates and eventual identification of the single or multiple VOCs that compose the Xcc scent signature is beyond this study, as is their individual concentrations necessary for canine detection. However, the realization of a scent signature composition that is at least in part sub-cellular opens a clear and exciting path for future explorations.

## 5. Conclusions

For millennia, humans have benefitted from application of the acute canine sense of smell to hunt, track, and find targets of importance. In this study, we demonstrated that canines can detect the Xcc phytobacterial pathogen of Asiatic citrus canker in simulated orchards, commercial orchards, and in a commercial packinghouse with high sensitivity, specificity, accuracy, and precision. Canines detected Xcc within 1–2 s of target interrogation time. Canines also alerted across a range of 1–12-week-old infections as well as across a range of pathogen prevalence with equal accuracy. Information theoretic analyses illustrate the diagnostic capacity of canines via their very high positive likelihood ratios for cases across pathogen prevalence at two orders of magnitude higher than values reported for other plant and human diseases. When trained to either Xcc-infected trees or Xcc axenic cultures, canines inherently detected the homologous and heterologous targets, suggesting they can detect Xcc directly rather than only volatiles produced by the host following infection. Canines were also able to detect the Xcc scent signature across a range of axenically cultured Xcc concentrations (10^4^ to 10^0^ = single cell) and even <1 bacterial cell, which implies that the scent signature is composed of bacterial cell volatile organic compound constituents or exudates that occur at concentrations many fold that of the bacterial cells. These findings indicate that Xcc cultures are a valuable surrogate targeting tool in the absence of infected plants. Results imply that canines can be trained and deployed as viable early detectors of Xcc across a diversity of environments and outperform the prevailing detection method, i.e., human visual detection.

## Figures and Tables

**Table 1:** Canines trained to detect *Xanthomonas citri* pv.*citri* (Xcc) scent signature and utilized in this study.

Canine	Breed	Trial(s)
Kimba	Belgian Malinois	Proof of concept studies/scent transfer unit
Tank	German Shepherd	Proof of concept studies/scent transfer unit
NDD-1	Beagle	Proof of concept studies/grapefruit seedlings
NDD-2	Beagle	Proof of concept studies/grapefruit seedlings
NDD-3	German Pointer	Proof of concept studies/grapefruit seedlings
NDD-4	Labrador retriever	Initial orchard detection/grapefruit leaves
Juice	Labrador retriever	Simulated orchards, Packinghouse, Commercial orchards, Spatial heterogeneity
Bady	German Shepherd	Lesion age, Lesion incidence, Abscised/senescing leaves, spatial heterogeneity
Maxi	German Shepherd	Lesion age, Lesion incidence, Abscised/senescing leaves, spatial heterogeneity
Mi	German Shepherd/Belgian Malinois	Direct bacterial detection, Bacterial dilution
Ti	German Shepherd	Direct bacterial detection, Bacterial dilution

**Table 2:** Latent class metrics for canine Xcc-infected tree detection in simulated 100-tree Duncan grapefruit citrus orchard by tree incidence accumulated over all the months tested.

Metric ^a^	Xcc-Infected Plant Incidence					
	2%	4%	5%	7%	10%	Overall
*n*	600	600	800	700	600	3300
TP	11	21	30	37	44	143
TN	587	573	758	647	540	3105
FP	1	3	3	4	0	11
FN	1	3	9	12	16	41
SEN	0.9167	0.8750	0.7692	0.7551	0.7333	0.7772
SPE	0.9983	0.9948	0.9961	0.9939	1.0000	0.9965
PPV	0.9167	0.8750	0.9091	0.9024	1.0000	0.9286
NPV	0.9983	0.9948	0.9883	0.9818	0.9712	0.9870
FPR	0.0017	0.0052	0.0039	0.0061	0.0000	0.0035
FDR	0.0833	0.1250	0.0909	0.0976	0.0000	0.0714
ACC	0.9967	0.9900	0.9850	0.9771	0.9733	0.9842
Month(s)	J,S,D	J,S,D	J,S,D,M	J,S,D,M	J,S,D	

^a^ Performance metrics as described in Section 2.3 above. Orchard grid consisted of 100 trees with the indicated Xcc-infected tree incidence. The grid was re-randomized between each replication, at each incidence level and interrogated by a single canine—“Juice”. Months tested: J = July, S = September, D = December, M = May.

**Table 3:** Latent class metrics for canine Xcc-infected tree detection in simulated 100-tree Duncan grapefruit citrus orchard for each month tested accumulated over all tree incidence levels tested.

Metric ^a^	Month of Test				
	July	September	December	May	Overall
*n*	900	1000	1000	400	3300
TP	42	40	44	17	143
TN	846	942	943	374	3105
FP	5	2	2	2	11
FN	7	16	11	7	41
SEN	0.8571	0.7143	0.8000	0.7083	0.7772
SPE	0.9941	0.9979	0.9979	0.9947	0.9965
PPV	0.8936	0.9524	0.9565	0.8947	0.9286
NPV	0.9918	0.9833	0.9885	0.9816	0.9870
FPR	0.0059	0.0021	0.0021	0.0053	0.0035
FDR	0.1064	0.0476	0.0435	0.1053	0.0714
ACC	0.9867	0.9820	0.9870	0.9775	0.9842

^a^ Performance metrics as described in Section 2.3 above. Orchard grid consisted of 100 trees with the indicated Xcc-infected tree incidence. The grid was re-randomized between each replication, at each incidence level and interrogated by a single canine—“Juice”.

**Table 4:** Latent class metrics for canine Xcc-infected tree detection in simulated 100-tree Duncan grapefruit citrus orchard by tree incidence accumulated for each canine tested.

	Bady							Maxi							Overall
	Xcc-Infected Plant Incidence							Xcc-Infected Plant Incidence							Both
Metrics ^a^	1%	2%	3%	4%	5%	6%	Overall	1%	2%	3%	4%	5%	6%	Overall	Canines
*n*	400	400	400	400	400	200	2200	400	400	400	400	300	200	2100	4300
TP	4	8	12	16	20	12	72	4	8	12	16	14	11	65	137
TN	396	388	387	380	380	186	2117	396	392	388	384	285	188	2033	4150
FP	0	4	1	4	0	2	11	0	0	0	0	0	0	0	11
FN	0	0	0	0	0	0	0	0	0	0	0	1	1	2	2
SEN	1.0000	1.0000	1.0000	1.0000	1.0000	1.0000	1.0000	1.0000	1.0000	1.0000	1.0000	0.9333	0.9167	0.9701	0.9856
SPE	1.0000	0.9898	0.9974	0.9896	1.0000	0.9894	0.9948	1.0000	1.0000	1.0000	1.0000	1.0000	1.0000	1.0000	0.9974
PPV	1.0000	0.6667	0.9231	0.8000	1.0000	0.857	0.8675	1.0000	1.0000	1.0000	1.0000	1.0000	1.0000	1.0000	0.9257
NPV	1.0000	1.0000	1.0000	1.0000	1.0000	1.000	1.0000	1.0000	1.0000	1.0000	1.0000	0.9965	0.9947	0.9990	0.9995
FPR	0.0000	0.0102	0.0026	0.0104	0.0000	0.011	0.0052	0.0000	0.0000	0.0000	0.0000	0.0000	0.0000	0.0000	0.0026
FDR	0.0000	0.3333	0.0769	0.2000	0.0000	0.143	0.1325	0.0000	0.0000	0.0000	0.0000	0.0000	0.0000	0.0000	0.0743
ACC	1.0000	0.9900	0.9975	0.9900	1.0000	0.9900	0.9950	1.0000	1.0000	1.0000	1.0000	0.9967	0.9950	0.9990	0.9970

^a^ Performance metrics as described in Section 2.3 above. Orchard grid consisted of 100 trees with the indicated Xcc-infected tree incidence. The grid was re-randomized between each replication, at each incidence level and interrogated by two canines—“Bady” and “Maxi”.

**Table 5:** Latent class metrics for canine detection of Xcc-infections of increasing age.

	Canine “Bady”						Canine “Maxi”						Canines
	Xcc-Lesion Age (wks)						Xcc-Lesion Age (wks)						Combined
Metric ^a^	1 + 2	3 + 4	6 + 7	9 + 10	12 + 13	Overall	1 + 2	3 + 4	6 + 7	9 + 10	12 + 13	Overall	Overall
*n*	500	500	500	500	500	2500	500	500	500	500	500	2500	5000
TP	20	18	18	18	18	92	19	20	19	20	19	97	189
TN	480	480	480	480	480	2400	480	480	480	480	480	2400	4800
FP	0	0	0	0	0	0	0	0	0	0	0	0	0
FN	0	2	2	2	2	8	1	0	1	0	1	3	11
SEN	1.0000	0.9000	0.9000	0.9000	0.9000	0.9200	0.9500	1.0000	0.9500	1.0000	0.9500	0.9700	0.9450
SPE	1.0000	1.0000	1.0000	1.0000	1.0000	1.0000	1.0000	1.0000	1.0000	1.0000	1.0000	1.0000	1.0000
PPV	1.0000	1.0000	1.0000	1.0000	1.0000	1.0000	1.0000	1.0000	1.0000	1.0000	1.0000	1.0000	1.0000
NPV	1.0000	0.9959	0.9959	0.9959	0.9959	0.9967	0.9979	1.0000	0.9979	1.0000	0.9979	0.9988	0.9977
FPR	0.0000	0.0000	0.0000	0.0000	0.0000	0.0000	0.0000	0.0000	0.0000	0.0000	0.0000	0.0000	0.0000
FDR	0.0000	0.0000	0.0000	0.0000	0.0000	0.0000	0.0000	0.0000	0.0000	0.0000	0.0000	0.0000	0.0000
ACC	1.0000	0.9960	0.9960	0.9960	0.9960	0.9968	0.9980	1.0000	0.9980	1.0000	0.9980	0.9988	0.9978

^a^ Performance metrics as described in Section 2.3 above. The simulated orchard grid consisted of 50 trees with two Xcc-infected trees of the indicated age group randomly placed with a population of 48 non-infected trees. The grid was re-randomized between each replication, at each incidence level and interrogated by two canines—“Bady” and “Maxi”.

**Table 6:** Latent class metrics for the effect of incidence of Xcc lesions on canine detection.

							Canines
	Lesions/Tree				Bady	Maxi	Combined
Metric ^a^	1	5	50	500	Overall	Overall	Overall
*n*	1880	1880	1880	1880	3760	3760	7520
TP	27	31	29	35	61	61	122
TN	1836	1836	1836	1836	3676	3668	7344
FP	4	4	4	4	4	12	16
FN	13	9	11	5	19	19	38
SEN	0.6750	0.7750	0.7250	0.8750	0.7625	0.7625	0.7625
SPE	0.9978	0.9978	0.9978	0.9978	0.9989	0.9967	0.9978
PPV	0.8710	0.8857	0.8788	0.8974	0.9385	0.8356	0.8841
NPV	0.9930	0.9951	0.9940	0.9973	0.9949	0.9948	0.9949
FPR	0.0022	0.0022	0.0022	0.0022	0.0011	0.0033	0.0022
FDR	0.1290	0.1143	0.1212	0.1026	0.0615	0.1644	0.1159
ACC	0.9910	0.9931	0.9920	0.9952	0.9939	0.9918	0.9928

^a^ Performance metrics as described in Section 2.3 above. The simulated orchard grid consisted of 100 trees with one Xcc-infected tree of each incidence level (1, 5, 50, and 500 lesions/tree) randomly placed within a population of 96 non-infected trees. The grid was re-randomized between each replication and interrogated by two canines—“Bady” and “Maxi”.

**Table 7:** Latent class metrics for canine detection of Xcc-infections in leaves decaying over time post abscission.

	Days Post-Abscission						Totals
Metric ^a^	0	1	2	5	13	27	Over Time
*n*	32	64	80	80	112	80	448
TP	8	15	8	9	16	0	56
TN	24	48	59	60	82	60	333
FP	0	0	1	0	2	0	3
FN	0	1	12	11	12	20	56
SEN	1.0000	0.9375	0.4000	0.4500	0.5714	0.0000	0.5000
SPE	1.0000	1.0000	0.9833	1.0000	0.9762	1.0000	0.9911
PPV	1.0000	1.0000	0.8889	1.0000	0.8889	NA	0.9492
NPV	1.0000	0.9796	0.8310	0.8451	0.8723	0.7500	0.8560
FPR	0.0000	0.0000	0.0167	0.0000	0.0238	0.0000	0.0089
FDR	0.0000	0.0000	0.1111	0.0000	0.1111	NA	0.0508
ACC	1.0000	0.9844	0.8375	0.8625	0.8750	0.7500	0.8683

^a^ Performance metrics as described in Section 2.3 above. Two canines “Bady” and “Maxi”, interrogated piles of Xcc-infected and non-infected decaying leaves at various assessment times post-leaf abscission. Leaf piles were continuously exposed to ambient conditions over time. Data were accumulated and combined over canines and replications. NA—metric could not be calculated due to a complete lack of canine detection by both canines, resulting in a calculated TP + FP = 0 value in the denominator.

**Table 8:** Latent class metrics for canine detection of Xcc-infections of various incidence in commercially packed boxes of red grapefruit.

	Incidence of Cartons Containing Xcc-Infected Fruit										
Metric ^a^	1	2	3	4	5	6	7	8	9	10	Total
*n*	100	700	400	400	100	200	200	300	200	200	2800
TP	1	12	11	11	3	9	10	15	14	12	98
TN	99	685	387	385	95	186	183	273	180	179	2652
FP	0	1	1	0	1	2	3	3	2	1	14
FN	0	2	1	4	1	3	4	9	4	8	36
SEN	1.0000	0.8571	0.9167	0.7333	0.7500	0.7500	0.7143	0.6250	0.7778	0.6000	0.7313
SPE	1.0000	0.9985	0.9974	1.0000	0.9896	0.9894	0.9839	0.9891	0.9890	0.9944	0.9947
PPV	1.0000	0.9231	0.9167	1.0000	0.7500	0.8182	0.7692	0.8333	0.8750	0.9231	0.8750
NPV	1.0000	0.9971	0.9974	0.9897	0.9896	0.9841	0.9786	0.9681	0.9783	0.9572	0.9866
FPR	0.0000	0.0015	0.0026	0.0000	0.0104	0.0106	0.0161	0.0109	0.0110	0.0056	0.0053
FDR	0.0000	0.0769	0.0833	0.0000	0.2500	0.1818	0.2308	0.1667	0.1250	0.0769	0.1250
ACC	1.0000	0.9957	0.9950	0.9900	0.9800	0.9750	0.9650	0.9600	0.9700	0.9550	0.9821

^a^ Performance metrics as described in Section 2.3 above. Incidence (1–10) indicates the number cartons containing Xcc-infected fruit placed in a grid of 100 commercially packed boxes each containing ≈50 red grapefruit.

**Table 9:** Latent class metrics for canine detection of Xcc-infections in commercial red grapefruit orchards in Indian River County, Florida.

			Orchard 2
Metric ^a^	Orchard 1	Orchard 2	Theoretical
*n*	445	345	345
TP	2	13	18
TN	436	325	325
FP	7	5	0
FN	0	2	2
SEN	1.0000	0.8667	0.9000
SPE	0.9842	0.9848	1.0000
PPV	0.2222	0.7222	1.0000
NPV	1.0000	0.9939	0.9939
FPR	0.0158	0.0152	0.0000
FDR	0.7778	0.2778	0.0000
ACC	0.9843	0.9797	0.9942

^a^ Performance metrics as described in Section 2.3 above. Orchard 1—10 rows of trees, 38 trees per row, 8 missing trees. Orchard 2—17 rows of trees, 29 trees per row, 47 missing trees. Orchard 2 (theoretical results for discussion) considers that the 5 FP detections were Xcc-infected but not detected by human visual survey.

**Table 10:** Latent class metrics for canine detection of Xcc-infected plants versus Xcc culture.

	Xcc-Infected Plants			Xcc Culture		
Metric ^a^	Mi	Ti	Overall	Mi	Ti	Overall
*n*	120	230	250	140	100	240
TP	10	13	23	11	9	20
TN	107	207	314	123	89	212
FP	1	0	1	3	1	4
FN	2	10	12	3	1	4
SEN	0.8333	0.5652	0.6571	0.7857	0.9000	0.8333
SPE	0.9907	1.0000	0.9968	0.9762	0.9889	0.9815
PPV	0.9091	1.0000	0.9583	0.7857	0.9000	0.8333
NPV	0.9817	0.9539	0.9632	0.9762	0.9889	0.9815
FPR	0.0093	0.0000	0.0032	0.0238	0.0111	0.0185
FDR	0.0909	0.0000	0.0417	0.2143	0.1000	0.1667
ACC	0.9750	0.9565	0.9629	0.9571	0.9800	0.9667

^a^ Performance metrics as described in Section 2.3 above. Two canines, Mi and Ti, were trained to detect Xcc-infected plants or Xcc cultures, respectively. Both canines interrogated both Xcc-infected plants or Xcc cultures to determine the effect of target used for training. Data represent the combination of two replications.

**Table 11:** Latent class metrics for canine detection of Xcc culture dilutions, detection threshold.

Metric ^a^	Canine Mi						
	Xcc Bacteria on Filter Pad Targets					Xcc Filtrate ^c^	*B. Meg* ^d^
	10^4^ cfu/mL = 26.4 cfu ^b^	10^2^ cfu/mL = 3.60 cfu	10^0^ cfu/mL = 0.27 cfu	10^−1^ cfu/mL = 0 cfu	10^−2^ cfu/mL = 0 cfu		
*n*	10	10	10	10	10	27	10
TP	1	1	1	1	1	1	0
TN	9	8	9	9	9	27	9
FP	0	1	0	0	0	0	0
FN	0	0	0	0	0	2	1
TPR	1.0000	1.0000	1.0000	1.0000	1.0000	0.3333	0.0000
SEN	1.0000	1.0000	1.0000	1.0000	1.0000	0.3333	0.0000
SPE	1.0000	0.8889	1.0000	1.0000	1.0000	1.0000	1.0000
PPV	1.0000	0.5000	1.0000	1.0000	1.0000	1.0000	0.0000
NPV	1.0000	1.0000	1.0000	1.0000	1.0000	0.9310	0.9000
FPR	0.0000	0.1111	0.0000	0.0000	0.0000	0.0000	0.0000
FDR	0.0000	0.5000	0.0000	0.0000	0.0000	0.0000	0.0000
ACC	1.0000	0.9000	1.0000	1.0000	1.0000	0.9333	0.9000
	**Canine Ti**						
*n*	10	20	10	10	10	30	20
TP	1	1	1	1	1	0	0
TN	9	18	9	9	9	27	18
FP	0	0	0	0	0	0	0
FN	0	1	0	0	0	3	2
TPR	1.0000	0.5000	1.0000	1.0000	1.0000	0.0000	0.0000
SEN	1.0000	0.5000	1.0000	1.0000	1.0000	0.0000	0.0000
SPE	1.0000	1.0000	1.0000	1.0000	1.0000	1.0000	1.0000
PPV	1.0000	1.0000	1.0000	1.0000	1.0000	0.0000	0.0000
NPV	1.0000	0.9474	1.0000	1.0000	1.0000	0.9000	0.9000
FPR	0.0000	0.0000	0.0000	0.0000	0.0000	0.0000	0.0000
FDR	0.0000	0.0000	0.0000	0.0000	0.0000	0.0000	0.0000
ACC	1.0000	0.9500	1.0000	1.0000	1.0000	0.9000	0.9000
	**Overall**						
*n*	20	30	20	20	20	60	30
TP	2	2	2	2	2	1	0
TN	18	26	18	18	18	54	27
FP	0	1	0	0	0	0	0
FN	0	1	0	0	0	5	3
TPR	1.0000	0.6667	1.0000	1.0000	1.0000	0.1667	0.0000
SEN	1.0000	0.6667	1.0000	1.0000	1.0000	0.1667	0.0000
SPE	1.0000	0.9630	1.0000	1.0000	1.0000	1.0000	1.0000
PPV	1.0000	0.6667	1.0000	1.0000	1.0000	1.0000	0.0000
NPV	1.0000	0.9630	1.0000	1.0000	1.0000	0.9153	0.9000
FPR	0.0000	0.0370	0.0000	0.0000	0.0000	0.0000	0.0000
FDR	0.0000	0.3333	0.0000	0.0000	0.0000	0.0000	0.0000
ACC	1.0000	0.9333	1.0000	1.0000	1.0000	0.9167	0.9000

^a^ Performance metrics as described in Section 2.3 above. Two canines, Mi and Ti were trained to detect Xcc-infected plants or Xcc cultures, respectively. Both dogs interrogated Xcc culture dilutions to determine the lower threshold of bacterial concentration that canines can detect. ^b^ Initial dilutions were prepared spectrophotometrically to 10^4^, 10^2^, 10^0^, 10^−1^ and 10^−2^ cfu/mL. Dilutions were presented to the canines and cultured the same day. Three days later, cultures were quantified and used to calculate the number of bacteria that were pipetted onto filter pads and presented to canines as targets, i.e., 26.4, 3.60, 0.27, 0, and 0 cfu, respectively. ^c^ **Xcc Filtrate**—bacterial culture filtrate prepared by filtration of ≈10^2^ cfu/mL culture through 0.2 µm filter to eliminate all bacterial cells. Filtrate was cultured and resulted in 0 cfu growth. ^d^ ***B. Meg*** = *Bacillius megaterium*, additional bacterium used as a negative control to ensure that canines were not reacting to random bacteria. Original dilution was 143 cfu/mL with ≈57 cfu/400 µL pipetted onto a sterile cotton filter disc.

## Supplementary Materials

The following are available online at https://www.mdpi.com/1099-4300/22/11/1269/s1, Video S1: Canine “Juice” surveilling simulated new planting of 100 young grapefruit trees. Trees with Xcc infection were randomized within rows. Note that the canine hesitates when it acquires the Xcc scent signature and the handler continues to pull on the leash in an attempt to dissuade the canine and thereby confirm the detection. Canine cannot be dissuaded to leave the TP Xcc-infected tree and eventually alerts by sitting next to the infected tree. Canines utilized in future trials were trained to alert (sit) immediately upon detection. Video S2: Canine “Juice” surveilling for 1–2 fruit with Xcc infections hidden within cardboard cartons of ≈50 commercially packed red grapefruit in a Florida packinghouse. Note canine hesitates when it acquires the Xcc scent signature and handler continues to pull on leash in an attempt to dissuade the canine and thereby confirm the detection. Canine cannot be dissuaded to leave the carton and eventually alerts by sitting next to a carton containing TP fruit with Xcc infections. Canines utilized in future trials were trained to alert (sit) immediately upon detection. Video S3: Canine “Mi” training to detect TP Xcc-infected tree randomized within a line of nine other TN trees. Canine alerts by sitting next to the TP Xcc-infected tree. Video S4: Canine “Mi” previously trained to detect Xcc-infected plants, correctly detects (alerts by sitting) on a metal can containing a TP cotton pad infused with axenic Xcc-culture suspension in PBS buffer in a line of cans with one TP and nine TN cans (with cotton pads infused with PBS buffer only) without prior training on Xcc culture. Video S5: Canine ‘Ti’ previously trained to detect TP cotton pads infused with axenic Xcc-culture suspension in PBS buffer, correctly detects (alerts by sitting) TP Xcc-infected tree in a line with one TP and nine TN trees without prior training on trees. Video S6: Canine “Ti” previously trained to detect TP cotton pads infused with axenic Xcc-culture suspension in PBS buffer, correctly detects (alerts by sitting) on a metal can containing a TP cotton pad infused with a 10^0^ cfu/mL concentration of Xcc bacteria (≅0.27 bacteria/pad), in a line of nine TN pads in cans. Indicates that canine may be acquiring the Xcc-scent signature and alerting on subcellular bacterial components. Note canine surveilled line of cans off leash. Video S7: Canine “Mi” interrogates a row of 11 suspect cans. The canine alerts by sitting next to TP can #6 containing a cotton pad infused with a 400 µL Xcc bacterial suspension in PBS buffer of 10^−2^ cfu/mL (≅0 bacteria/pad, subsequent determined via culturing). Remaining 10 cans contained TN cotton pads infused with 400 µL PBS buffer only. Handler rewards canine for correct detection with a few moments of play with a hard rubber ‘kong’ toy. Detection of bacterial concentration containing less than one bacteria indicates canine recognizes a scent signature composed of subcellular bacterial components. Video S8: Canine ‘Ti’ interrogates a row of 11 suspect cans. The canine alerts by sitting next to TP can #6 containing a cotton pad infused with a 400 µL Xcc bacterial suspension in PBS buffer of 10^−2^ cfu/mL (≅0 bacteria/pad, subsequent determined via culturing). Remaining 10 cans contained TN cotton pads infused with 400 µL PBS buffer only. Handler rewards canine for correct detection with a few moments of play with a hard rubber “kong” toy. Detection of bacterial concentration containing less than one bacteria indicates that the canine recognizes a scent signature composed of subcellular bacterial components.
